# Supplementary material for: Identification of Freezing-Responsive microRNAs and Their Targets in Chinese Jujube by Small RNA and Degradome Sequencing
Source: Int J Mol Sci. 2026 Sep 4;27(17):7912. doi: 10.3390/ijms27177912 (PMC13566890; doi:10.3390/ijms27177912)
Supplement: Supplementary file 1 [file ijms-27-07912-s001.zip › Supplementary Material S2/novel_miR_11.pdf]

Provisional ID	:	novel_miR_11
Score total	:	202245.3
Score for star read(s)	:	3.9
Score for read counts	:	202239.1
Score for mfe	:	0.7
Score for randfold	:	1.6
Score for cons. seed	:	
Total read count	:	396695
Mature read count	:	396569
Loop read count	:	3
Star read count	:	123

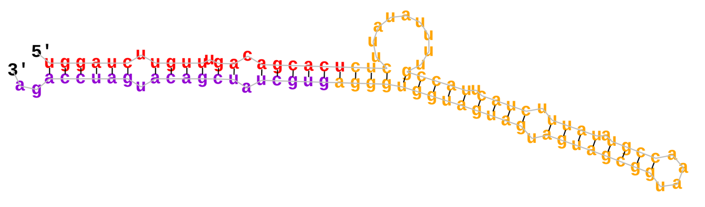

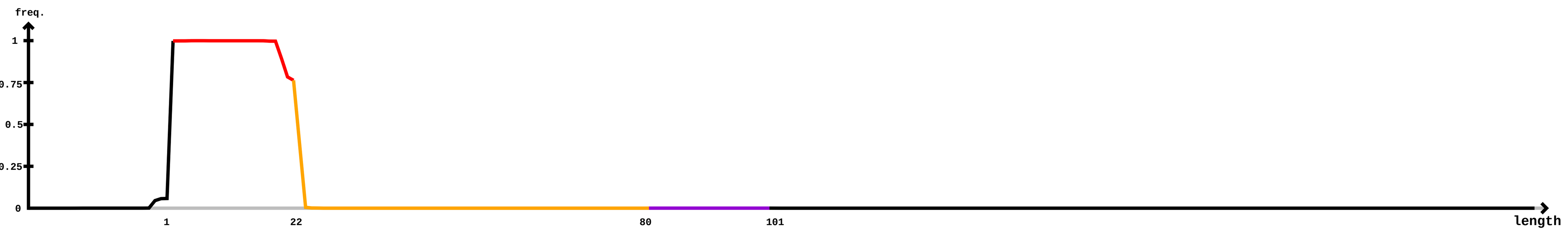

Mature Star

```
5'- uccaucuuguucguuuacccucuggaucuuguuugacagcacucucuuauauuuugccauucaucuuuauaugccaaauggcgaugaugaugauggugggagugcuaucgacaugauccagaaggugcagucgaauaagauacagagggaccacccacaguaccuacucucguuacaaucguauucuucuccucacuuauuauuauuauuauuauuauuccgaucauuuucuuuuaaucuauugaag -3' obs
    uccaucuuguucguuuacccucuggaucuuguuugacagcacucucuuauauuuugccauucaucuuuauaugccaaauggcgaugaugaugaugguggagugcuaucgacaugauccagaaggugcagucgaauaagauacagagggaccacccacaguaccuacucucguuacaaucguauucuucuccucacuuauuauuauuauuauuauuauuccgaucauuuucuuuuaaucuauugaag     exp
    ...(((((((((((((((.((((((((.((((.((.(((((((((.........(((((.((((.((((.((((....)))))))).))))))))))))))))).))))))).)))))))).)))).)).))))))))))(((((((((........)).))..)))).))...........     reads   mm   sample
    .ccaucuuguucguuuacc............     1   0   LT-1
    .ccaucuuguucguuuacccucugg......     5   0   LCK-3
    .ccaucuuguucguuuacccucuggauc...     1   0   LT-3
    ...aucuuguucguuuacccucugga.....     2   0   JCK-1
    .....cuuguucguuuacccucuggauc...     4   0   LT-2
    ......uuguucguuuaccUucuggauc...     6   1   JCK-2
    ......uuguucguuuaccUucuggauc...     3   1   JCK-3
    ......uuguucguuuaccUucuggauc...     2   1   LT-2
    ......uuguucguuuaccUucuggauc...     2   1   JCK-1
    .......uguucguuuaccUucuggauc...     12  1   LT-1
    .......uguucguuuaccUucuggauc...     12  1   LT-3
    .......uguucguuuaccUucuggauc...     10  1   JCK-1
    .......uguucguuuacccucuggauc...     1   0   JCK-1
    .......uguucguuuaccUucuggauc...     30  1   JCK-3
    .......uguucguuuaccUucuggauc...     3   1   LT-2
    .......uguucguuuacccucuggauc...     2   0   JCK-3
    .......uguucguuuaccUucuggauc...     31  1   JCK-2
    .......uguucguuuacccucuggauc...     1   0   JCK-2
    ..........ucguuuacccucuggaucuug...  1   0   LT-2
    ............guuuacccucuggaucuuguuugaca...  2   0   LCK-3
    .............uuuacccucuggaucuuguA...  1   1   JCK-1
    .............uuuacccucuggaucuuguuu...  2   0   JCK-3
    .............uuuacccucuggaucuuguuuga...  1   0   JCK-1
    ...............uacccucuggaucuuguuugacagc...  2   0   JCK-2
    ................Ccccucuggaucuuguuuga...  1   1   LT-1
    ................acccucuggaucuuguuugacagc...  2   0   JCK-2
    ................acccucuggaucuuguuugacagca...  2   0   JCK-2
    ................acccucuggaucuuguuugacagca...  2   0   JCK-3
    .................cccucuggaucuuguuugacagc...  1   0   LT-3
    ..................ccucuggaucuuguuugac...  3   0   JCK-2
    ..................ccucuggaucuuguuugaca...  2   0   JCK-3
    ..................ccucuggaucuuguuugaca...  2   0   JCK-1
    ..................ccucuggaucuuguuugacag...  1   0   JCK-1
```

Mature **Star**

uccaucuuguucguuuacccucuggaucuuguuugacagcacucucuuauauuuugccauucaucuuuauaugccaaauggcgaugaugaugauggugggagugcuaucgacaugauccagaaggugcagucgaauaagauacagagggaccacccacaguaccuacucucguuacaaucguauucuucuccucacuuauuauuauuauuauuauuauuccgaucauuuucuuuuaaucuauugaag

ccucuggaucuuguuugacag	1	0	JCK-2
ccucuggaucuuguuugacag	1	0	JCK-3
ccucuggaucuuguuugacag	4	0	LCK-3
ccucuggaucuuguuugacagc	6	0	LCK-3
ccucuggaucuuguuugacagc	9	0	LT-3
ccucuggaucuuguuugacagc	32	0	JCK-2
ccucuggaucuuguuugacagc	21	0	JCK-1
ccucuggaucuuguuugacagc	3	0	LT-2
ccucuggaucuuguuugacagc	35	0	JCK-3
ccucuggaucuuguuugacagc	6	0	LT-1
ccucuggaucuuguuugacagcU	2	1	JCK-1
ccucuggaucuuguuugacagcU	1	1	JCK-3
ccucuggaucuuguuugacagcU	4	1	JCK-2
cucuggaucuuguuugac	6	0	LCK-3
cucuggaucuuguuugac	13	0	JCK-2
cucuggaucuuguuugac	13	0	LT-2
cucuggaucuuguuugac	10	0	JCK-3
cucuggaucuuguuugac	13	0	LT-3
cucuggaucuuguuugac	4	0	JCK-1
cucuggaucuuguuugac	8	0	LT-1
cucuggaucuuguuugaU	2	1	JCK-3
cucuggCucuuguuugac	1	1	JCK-2
cucuggaucuuguuugaca	41	0	JCK-2
cuNggaucuuguuugaca	1	1	LT-2
cucuggaucuuguuugacU	7	1	JCK-2
cucuggaucuuguuugaca	18	0	JCK-3
cucuggaucuuguuugaca	62	0	LT-2
cucuggCucuuguuugaca	1	1	LCK-1
cucuggaucuuguuugaca	65	0	LT-3
cucuggaucuuguuugacU	2	1	LT-1
cucuggaucuuguuugaca	42	0	LCK-1
cucuggCucuuguuugaca	1	1	JT-1
cucuggaucuuguuugaca	21	0	JT-3
cucuggaucuuguuugacC	1	1	LT-1
cucuggaucuuguuugaUa	1	1	JCK-3
cucuggaucuuguuugacU	6	1	LT-2
cucuggaucuuguuugaca	27	0	LT-1
cucuggaucuuguuugaca	78	0	LCK-3
cucuggaucuuguuugacU	1	1	LT-3
cucuggaucuuguuugaca	28	0	JT-1
cucuggaucuuguuugaca	18	0	JT-2
cucuggaucuuguuugacU	14	1	LCK-3
cucuggaucuuguuugaca	20	0	JCK-1
cucuggaucuuguuugaca	29	0	LCK-2
cucuggaucuuguuugacU	6	1	JCK-3
cucuggaucuuguuugaUa	1	1	JCK-2
cucuggaucuuguuugacag	5	0	JCK-1
cucuggaucuuguuugacag	7	0	LT-3
cucuggaucuuguuugacag	23	0	LCK-3
cucuggaucuuguuugacag	6	0	JCK-3
cucuggaucuuguuugacag	4	0	JCK-2
cucuggaucuuguuugacag	12	0	LT-2
cucuggaucuuguuugacaU	2	1	LT-1
cucuggaucuuAuuugacag	1	1	LT-2
cucuggaucuuguuugacaU	3	1	LT-3
cucuggaucuuguuugacag	7	0	LT-1
cucuggaucuuguuugacag	14	0	JT-2
cucuggaucuuguuugacag	15	0	JT-3
cucuggaucuuguCugacagc	1	1	JCK-3
cucCggaucuuguuugacagc	1	1	LT-2
cucuggaucuuguuugacagc	2088	0	JCK-3
cucuggaucuuguuugaUagc	2	1	JCK-2
cucuggaucuuguuugacCgc	2	1	JCK-3
cucuggCucuuguuugacagc	1	1	JCK-1
cucuggaucuuguuugacagc	62	0	JT-2
cucuAgaucuuguuugacagc	1	1	JCK-1
cucuggaucuCguuugacagc	1	1	LT-1
cucuggaucuuguuugCcagc	1	1	JCK-3
cucuggaucuuguuugacagc	1680	0	JCK-1
cucuggaucuuguuugacagc	292	0	LCK-2

```
                      Mature                                                                          Star
uccaucuuguucguuuacccucuggaucuuguuugacagcacucucuuauauuuugccauucaucuuuauaugccaaauggcgaugaugaugauggugggagugcuaucgacaugauccagaaggugcagucgaauaagauacagagggaccacccacaguaccuacucucguuacaaucguauucuucucccucacuuauuauuauuauuauuauuauuauuccgaucauuucuuuuaaucuauugaag
...................cucuggaucuuguuugacCgc...................................................................................................................................................................................................   2     1     LT-1
...................cucuAgaucuuguuugacagc...................................................................................................................................................................................................   4     1     LT-2
...................cucuggaucuuguuuUacagc...................................................................................................................................................................................................   1     1     JCK-2
...................cucuggaucuugCuugacagc...................................................................................................................................................................................................   1     1     JCK-2
...................cucCggaucuuguuugacagc...................................................................................................................................................................................................   2     1     JCK-2
...................cucuggaucuuguuugacagA...................................................................................................................................................................................................   3     1     LT-2
...................cucuggaucuuNuuugacagc...................................................................................................................................................................................................   3     1     JCK-1
...................cNcuggaucuuguuugacagc...................................................................................................................................................................................................   1     1     JCK-3
...................cuNuggaucuuguuugacagc...................................................................................................................................................................................................   2     1     LT-2
...................cucuggaucuuAuuugacagc...................................................................................................................................................................................................   2     1     JCK-2
...................cucuggaucuuguuugacagc...................................................................................................................................................................................................   90    0     JT-3
...................cucuUgaucuuguuugacagc...................................................................................................................................................................................................   1     1     LT-2
...................cucugAaucuuguuugacagc...................................................................................................................................................................................................   1     1     JCK-3
...................cucCggaucuuguuugacagc...................................................................................................................................................................................................   1     1     LT-1
...................cNcuggaucuuguuugacagc...................................................................................................................................................................................................   1     1     LT-3
...................cuUuggaucuuguuugacagc...................................................................................................................................................................................................   2     1     JCK-1
...................cucCggaucuuguuugacagc...................................................................................................................................................................................................   3     1     LT-3
...................cucuggaucuCguuugacagc...................................................................................................................................................................................................   1     1     LCK-3
...................cucuggaCcuuguuugacagc...................................................................................................................................................................................................   2     1     LT-1
...................cucuggaucuuguuugacCgc...................................................................................................................................................................................................   2     1     JCK-1
...................cucuggaucuuguuugacCgc...................................................................................................................................................................................................   4     1     LT-3
...................cucuAgaucuuguuugacagc...................................................................................................................................................................................................   3     1     LT-3
...................cucuggaucuuguuugCcagc...................................................................................................................................................................................................   3     1     JCK-2
...................cucuggaucuuguuugacCgc...................................................................................................................................................................................................   2     1     JCK-2
...................cucuggaucuuguuugCcagc...................................................................................................................................................................................................   5     1     LT-3
...................cCcuggaucuuguuugacagc...................................................................................................................................................................................................   1     1     LCK-2
...................cucugAaucuuguuugacagc...................................................................................................................................................................................................   1     1     LT-3
...................cucuggCucuuguuugacagc...................................................................................................................................................................................................   4     1     JCK-3
...................cucuggaucuuguuugacGgc...................................................................................................................................................................................................   1     1     LT-3
...................cucuggaucuuguuugacCgc...................................................................................................................................................................................................   2     1     LT-2
...................cuGuggaucuuguuugacagc...................................................................................................................................................................................................   1     1     LT-2
...................cucuggaucCuguuugacagc...................................................................................................................................................................................................   1     1     JCK-2
...................cucuggaucuuguuugacagU...................................................................................................................................................................................................   2     1     LT-3
...................cucuggCucuuguuugacagc...................................................................................................................................................................................................   11    1     LT-1
...................cucuggaucuuguuugCcagc...................................................................................................................................................................................................   7     1     LT-2
...................cucuggaucuuguuugGcagc...................................................................................................................................................................................................   1     1     JCK-3
...................cucuggaucuuguuugaUagc...................................................................................................................................................................................................   1     1     LT-3
...................cucuggCucuuguuugacagc...................................................................................................................................................................................................   11    1     JCK-2
...................cuAuggaucuuguuugacagc...................................................................................................................................................................................................   2     1     JCK-3
...................cucuggCucuuguuugacagc...................................................................................................................................................................................................   20    1     LT-2
...................cucuggauNuuguuugacagc...................................................................................................................................................................................................   2     1     JCK-3
...................cucuggaucuuAuuugacagc...................................................................................................................................................................................................   1     1     LT-1
...................cucuggaucuuguuugaGagc...................................................................................................................................................................................................   1     1     JCK-1
...................cucuggaucuuguuugacagc...................................................................................................................................................................................................   275   0     LCK-1
...................cuUuggaucuuguuugacagc...................................................................................................................................................................................................   3     1     JCK-3
...................cucuggaucuCguuugacagc...................................................................................................................................................................................................   2     1     LT-2
...................cucCggaucuuguuugacagc...................................................................................................................................................................................................   2     1     JCK-3
...................cucugUaucuuguuugacagc...................................................................................................................................................................................................   1     1     LT-1
...................cNcuggaucuuguuugacagc...................................................................................................................................................................................................   1     1     LCK-1
...................cucuggaucuuguuugacagc...................................................................................................................................................................................................   66    0     JT-1
...................cucuggCucuuguuugacagc...................................................................................................................................................................................................   11    1     LT-3
...................cucuggauAuuguuugacagc...................................................................................................................................................................................................   1     1     LT-1
...................cCcuggaucuuguuugacagc...................................................................................................................................................................................................   1     1     LT-1
...................cucuggCucuuguuugacagc...................................................................................................................................................................................................   1     1     LCK-2
...................cucuAgaucuuguuugacagc...................................................................................................................................................................................................   1     1     JCK-2
...................cucuggaucuuguuugacCgc...................................................................................................................................................................................................   2     1     LCK-3
...................cucuggCucuuguuugacagc...................................................................................................................................................................................................   4     1     LCK-3
...................cucNggaucuuguuugacagc...................................................................................................................................................................................................   2     1     JCK-1
...................cNcuggaucuuguuugacagc...................................................................................................................................................................................................   4     1     JCK-2
...................cucGggaucuuguuugacagc...................................................................................................................................................................................................   1     1     JCK-1
...................cucugNaucuuguuugacagc...................................................................................................................................................................................................   1     1     LCK-1
...................cCcuggaucuuguuugacagc...................................................................................................................................................................................................   1     1     JCK-2
...................cucuggaucuuguCugacagc...................................................................................................................................................................................................   1     1     JCK-2
...................cNcuggaucuuguuugacagc...................................................................................................................................................................................................   2     1     JCK-1
...................cucuggaucuuguuugacagc...................................................................................................................................................................................................   1379  0     LCK-3
...................cucugAaucuuguuugacagc...................................................................................................................................................................................................   3     1     LT-2
...................cucuggaucCuguuugacagc...................................................................................................................................................................................................   1     1     LT-1
...................cucuggaucuuUuuugacagc...................................................................................................................................................................................................   1     1     JCK-3
...................cucuggaucuuguuugacagc...................................................................................................................................................................................................   1675  0     LT-2
...................cucuggaucuuguuugacagc...................................................................................................................................................................................................   3001  0     LT-3
```

```
                     Mature                                                                   Star
uccaucuuguucguuuacccucuggaucuuguuugacagcacucucuuauauuuugccauucaucuuuauaugccaaauggcgaugaugaugaugguggggagugcuaucgacaugauccagaaggugcagucgaauaagauacagagggaccacccacaguaccuacucucguuacaaucguauucuucuccucacuuauuauuauuauuauuauuauuauuccgaucauuuucuuuuaaucuauugaag
...................cCcuggaucuuguuugacagc..................................................................................................................................................................................................................   1      1    LCK-3
...................cucuggaucuuguuugCcagc..................................................................................................................................................................................................................   4      1    JCK-1
...................cucuggaucuuguuuAacagc..................................................................................................................................................................................................................   1      1    LCK-3
...................cucuggaucuuguuuAacagc..................................................................................................................................................................................................................   1      1    LT-1
...................cucuggaucuuguuugaUagc..................................................................................................................................................................................................................   2      1    JCK-3
...................cucuggaucuuUuuugacagc..................................................................................................................................................................................................................   1      1    LT-1
...................cucuUgaucuuguuugacagc..................................................................................................................................................................................................................   1      1    LT-3
...................cucuggaucuuguuugacagc..................................................................................................................................................................................................................   2907   0    JCK-2
...................cucuAgaucuuguuugacagc..................................................................................................................................................................................................................   1      1    LCK-3
...................cucuggauUuuguuugacagc..................................................................................................................................................................................................................   1      1    LT-1
...................cucuggaucuuguuugacagU..................................................................................................................................................................................................................   1      1    JCK-3
...................cucuggaucuuguuugacaNc..................................................................................................................................................................................................................   4      1    JCK-1
...................cGcuggaucuuguuugacagc..................................................................................................................................................................................................................   1      1    LT-2
...................cucuggCucuuguuugacagc..................................................................................................................................................................................................................   2      1    LCK-1
...................cucuggaucuuguuugCcagc..................................................................................................................................................................................................................   9      1    LT-1
...................cucuggaucuuguuugCcagc..................................................................................................................................................................................................................   2      1    LCK-3
...................cCcuggaucuuguuugacagc..................................................................................................................................................................................................................   2      1    LT-2
...................cucuggaucuuguuugacagc..................................................................................................................................................................................................................   3319   0    LT-1
...................cucuggaucuuAuuugacagc..................................................................................................................................................................................................................   1      1    LT-3
...................cucuggaucuuguuugacagcC.................................................................................................................................................................................................................   2      1    LT-2
...................cucuggaucuuguuugacagcU.................................................................................................................................................................................................................   13     1    JT-2
...................cucuggaucuuguuugacagca.................................................................................................................................................................................................................   27     0    LT-1
...................cucuggaucuuguuuNacagca.................................................................................................................................................................................................................   1      1    JCK-3
...................cucuggaucuuguuugacagca.................................................................................................................................................................................................................   5      0    LCK-3
...................cucuggaucuuguuugacagca.................................................................................................................................................................................................................   1      0    JT-2
...................cucuggaucuuguuugacagca.................................................................................................................................................................................................................   10     0    JT-3
...................cucuggaucuuguuugacagcU.................................................................................................................................................................................................................   55     1    JCK-2
...................cucuggaucuuguuugacagcU.................................................................................................................................................................................................................   66     1    LT-1
...................cucuggaucuuguuugacagcC.................................................................................................................................................................................................................   2      1    LT-3
...................cucuggaucuuguuugacagcC.................................................................................................................................................................................................................   1      1    JCK-2
...................cucuggaucuuguuugacagcU.................................................................................................................................................................................................................   29     1    JCK-3
...................cucuggaucuuguuugacagca.................................................................................................................................................................................................................   22     0    LT-3
...................cucuggaucuuguuugacagca.................................................................................................................................................................................................................   19     0    LT-2
...................cucuggaucuuguuugacagcG.................................................................................................................................................................................................................   1      1    JT-2
...................cucuggaucuuguuugacagca.................................................................................................................................................................................................................   72     0    JCK-1
...................cucuggaucuuguuugacagcU.................................................................................................................................................................................................................   84     1    LCK-3
...................cucuggaucuuguuugacagcU.................................................................................................................................................................................................................   14     1    JT-3
...................cucuggaucuugCuugacagca.................................................................................................................................................................................................................   1      1    JCK-2
...................cucuggaucuuguuugacagca.................................................................................................................................................................................................................   117    0    JCK-2
...................cucuggaucuuguuugacagca.................................................................................................................................................................................................................   87     0    JCK-3
...................cucuggaucuuguuugacagcU.................................................................................................................................................................................................................   25     1    JCK-1
...................cucuggaucuuguuugacagcU.................................................................................................................................................................................................................   69     1    LT-3
...................cucuggaucuuguuugacagcU.................................................................................................................................................................................................................   47     1    LT-2
...................cucuggaucuuguuugacagcC.................................................................................................................................................................................................................   2      1    LT-1
...................cucuggaucuuguuugacagcac................................................................................................................................................................................................................   1      0    LT-3
...................cucuggaucuuguuugacagcac................................................................................................................................................................................................................   1      0    LT-1
...................cucuggaucuuguuugacagcacN...............................................................................................................................................................................................................   1      1    LT-2
...................cucuggaucuuguuugacagcacu...............................................................................................................................................................................................................   1      0    LT-2
....................ucuggaucuuguuugacU....................................................................................................................................................................................................................   1      1    JCK-1
....................ucuggaucuuguuugaca....................................................................................................................................................................................................................   9      0    JT-1
....................ucuggaucuuguuugacU....................................................................................................................................................................................................................   6      1    LT-1
....................ucuggaucuuguuugacU....................................................................................................................................................................................................................   9      1    LCK-3
....................ucuggaucuuguuugacU....................................................................................................................................................................................................................   2      1    LT-3
....................ucuggaucuuguuugaca....................................................................................................................................................................................................................   41     0    LT-2
....................ucuggaucuuguuugaca....................................................................................................................................................................................................................   30     0    JT-2
....................ucuggaucuuguuugaca....................................................................................................................................................................................................................   3      0    LCK-2
....................ucuggaucuuguuugaca....................................................................................................................................................................................................................   3      0    LCK-1
....................ucuggaucuuguuugacU....................................................................................................................................................................................................................   1      1    JCK-2
....................ucuggaucuuguuugaca....................................................................................................................................................................................................................   15     0    JCK-3
....................ucuggaucuuguuugaca....................................................................................................................................................................................................................   33     0    JCK-2
....................ucuggaucuuguuugaca....................................................................................................................................................................................................................   28     0    LT-1
....................ucuggaucuuguuugaca....................................................................................................................................................................................................................   35     0    JT-3
....................ucuggaucuuguuugaca....................................................................................................................................................................................................................   79     0    LCK-3
....................ucuggaucuuguuugaca....................................................................................................................................................................................................................   26     0    LT-3
....................ucuggaucuuguuugaca....................................................................................................................................................................................................................   25     0    JCK-1
....................ucuggaucuuguuugacag...................................................................................................................................................................................................................   2      0    LT-1
....................ucuggaucuuguuugacagA..................................................................................................................................................................................................................   1      1    LT-3
....................ucuggaucuuguuuNacagc..................................................................................................................................................................................................................   1      1    LCK-1
....................ucuggaucuuguuugacagc..................................................................................................................................................................................................................   63     0    LCK-1
....................ucuggaucuuguuugacaUc..................................................................................................................................................................................................................   1      1    LT-1
```

Mature   Star

uccaucuuguucguuuacccucuggaucuuguuugacagcacucucuuauauuuugccauucaucuuuauaugccaaauggcgaugaugaugauggugggagugcuaucgacaugauccagaaggugcagucgaauaagauacagagggaccacccacaguaccuacucucguuacaaucguauucuucuccucacuuauuauuauuauuauuauuauuccgaucauuuucuuuuaaucuauugaag

....................ucuggCucuuguuugacagc	2	1	LT-2
....................ucuggaucuuguuugacagA	1	1	JCK-1
....................ucuggaucuuguuugacagc	35	0	JCK-3
....................ucuggaucuuguuuUacagc	1	1	LCK-2
....................ucuggaucuuguuugacagA	1	1	JCK-3
....................ucuggaucuuguuugacagc	41	0	LCK-3
....................ucuggaucuuguuugacagc	127	0	LT-3
....................ucuggaucuuguuugacagc	116	0	LT-2
....................ucuggaucuuguuugacagc	55	0	JCK-2
....................ucuggCucuuguuugacagc	1	1	LCK-1
....................ucuggaucuuguuugacagc	75	0	LCK-2
....................ucuggaucuuguuugacagc	107	0	LT-1
....................uGuggaucuuguuugacagc	1	1	LCK-1
....................ucuggaucuuguuugacagc	29	0	JCK-1
....................ucuggaucuuguuugacagca	299	0	LT-1
....................ucuggaucuuguuugacaUca	3	1	JCK-3
....................ucuggaucuuguuugacagca	267	0	LT-3
....................ucuggaucuuguuugCcagca	1	1	LT-1
....................uUuggaucuuguuugacagca	12	1	JCK-2
....................ucuggaucuugGuugacagca	2	1	LT-1
....................ucuggaucuuguuugacagca	9	0	LCK-1
....................ucuggaucuuguuugacGgca	2	1	JCK-1
....................ucuggaucuuguuugacaUca	2	1	JCK-1
....................ucuggauAuuguuugacagca	1	1	JCK-2
....................ucuggaucuuguuugacagcU	3	1	JCK-2
....................ucuggaucuuguuugGcagca	1	1	LCK-3
....................ucuggauUuuguuugacagca	1	1	JCK-2
....................ucuggCucuuguuugacagca	1	1	JCK-2
....................ucuggaucuuguuugacagca	11	0	JT-2
....................ucuggaucuuguuugacagca	12	0	JT-3
....................ucuggaucuuguuugacagcC	1	1	LCK-3
....................ucuggaucuuguuugacagca	17	0	LCK-2
....................ucuggaucuuguuugacagcU	6	1	LT-1
....................ucuggaucuuguuugacagcU	3	1	JCK-3
....................ucuggaucuuguuugacagca	628	0	JCK-1
....................ucuggaucuuguuugacagca	145	0	LCK-3
....................ucuggaucuuguuugUcagca	1	1	JCK-3
....................ucuggaucuuguuugacaUca	5	1	JCK-2
....................ucuggaucuuguuugUcagca	1	1	JCK-2
....................uUuggaucuuguuugacagca	1	1	JCK-1
....................ucuggaucuuguuugCcagca	1	1	JCK-1
....................ucuggaucuuguuugGcagca	1	1	LT-2
....................ucuggaucuuguuugacagca	832	0	JCK-3
....................ucuggaucuuguuugacagcU	7	1	LT-3
....................ucuggaucCuguuugacagca	1	1	JCK-2
....................uUuggaucuuguuugacagca	8	1	JCK-3
....................ucuggaucuuguuugacagca	195	0	LT-2
....................ucuggaucuuguuugacagca	1097	0	JCK-2
....................ucCggaucuuguuugacagca	1	1	JCK-1
....................ucuggaucuuguuugacagcU	9	1	LCK-3
....................ucuggaucuuguuugacagca	29	0	JT-1
....................ucuggaucuuguuugacagcN	1	1	JCK-1
....................ucuggaucuuguuugacagNa	2	1	JCK-1
....................ucuggaucuugGuugacagca	1	1	JCK-1
....................ucuNgaucuuguuugacagca	1	1	JCK-1
....................ucuggaucuuguuugacagUa	1	1	JCK-2
....................ucuggaucuuguuugacagcU	6	1	JCK-1
....................uNuggaucuuguuugacagca	1	1	JCK-1
....................ucCggaucuuguuugacagca	1	1	JCK-2
....................ucuggaucuuguuugCcagca	2	1	LT-3
....................ucuggaucuCguuugacagca	1	1	LT-3
....................ucuggaucuuguuugacagcU	14	1	LT-2
....................ucuggaucuuguuugacagcac	17	0	LT-2
....................ucuggaucuuguuugacagcaU	2	1	LT-2
....................ucuggaucuuguuugacagcac	19	0	LCK-3
....................ucuggaucuuguuugacagcac	50	0	JCK-2
....................ucuggaucuuguuugacagcac	22	0	JCK-3
....................ucuggaucuuguuugacagcaU	1	1	LT-1
....................ucuggaucuuguuugacagcac	24	0	LT-3
....................ucuggaucuuguuugacagcac	27	0	LT-1

Mature | **Star**

uccaucuuguucguuuacccucuggaucuuguuugacagcacucucuuauauuuugccauucaucuuuauaugccaaauggcgaugaugaugaugggga gugcuaucgacaugauccaga aggugcagucgaauaagauacagagggaccacccacaguaccuacucucguuacaaucguauucuucuccucacuuauuauuauuauuauuauuauuccgaucauuucuuuuaaucuauugaag

....................ucuggaucuuguuugCcagcac	1	1	LT-2
....................ucuggaucuuguuugacagcac	34	0	JCK-1
....................ucuggaucuuguuugacagcacu	2	0	JCK-3
....................ucuggaucuuguuugacagcacu	2	0	JCK-1
....................ucuggaucuuguuugacagcacu	5	0	JCK-2
....................ucuggaucuuguuugacagcacu	3	0	LT-1
....................ucuggaucuuguuugacagcaUu	1	1	LT-3
....................ucuggaucuuguuugacagcacuc	2	0	LT-3
....................ucuggaucuuguuugacagcacuc	1	0	JCK-2
....................ucuggaucuuguuugacagcacuc	1	0	JCK-1
....................ucuggaucuuguuugacagcacuc	1	0	JCK-3
....................ucuggaucuuguuugacagcacucu	2	0	LT-1
.....................cuggaucuuguuugacagc	1	0	JCK-1
.....................cuggaucuuguuugacagc	7	0	JCK-2
.....................cuggaucuuguuugacagc	13	0	LT-2
.....................cuggaucuuguuugacagc	5	0	LT-1
.....................cuggaucuuguuugacagc	2	0	JCK-3
.....................cuggaucuuguuugacagc	5	0	LCK-3
.....................cuggaucuuguuugacagc	1	0	LT-3
.....................cuggaucuuguuugacagca	58	0	JCK-1
.....................cuggaucuuguuugacagca	15	0	LT-3
.....................cuggaucuuguuugacagca	11	0	LT-2
.....................cuggaucuuguuugacagcU	1	1	JCK-3
.....................cuggaucuuguuugacagcU	1	1	JCK-2
.....................cuggaucuuguuugacagca	61	0	JCK-2
.....................cuggaucuuguuugacagca	12	0	LCK-3
.....................cuggaucuuguuugacagca	51	0	JCK-3
.....................cuggaucuuguuugacagcU	5	1	LT-2
.....................cuggaucuuguuugacagcU	4	1	JT-2
.....................cuggaucuuguuugacagca	9	0	LT-1
.....................cuggaucuuguuugacagcU	1	1	LT-3
.....................cuggaucuuguuugCcagca	1	1	JCK-1
.....................cuggaucuuguuugacagcU	4	1	JT-3
.....................cuggaucuuguuugacagcac	1	0	LCK-3
.....................cuggaucuuguuugacagcac	3	0	LT-3
.....................cuggaucuuguuugacagcac	4	0	LT-2
.....................cuggaucuuguuugacagcacu	22	0	JCK-3
.....................cuggaucuuguuugacagcaUu	2	1	JCK-3
.....................cuggaucuuguuugacagcacu	7	0	LT-2
.....................cuggaucuuguuugacagcacu	15	0	LCK-3
.....................cuggaucuuguuugacagcaUu	1	1	JCK-2
.....................cuggaucuuguuugacagcacu	13	0	LT-3
.....................cuggaucuuguuugacagcacu	46	0	JCK-2
.....................cuggaucuuguuugacagcacu	17	0	JCK-1
.....................cuggaucuuguuugacagcacu	4	0	LT-1
.....................cuggaucuuguuugacagcacuc	1	0	JCK-1
.....................cuggaucuuguuugacagcacuc	1	0	JCK-2
.....................cuggaucuuguuugacagcacuc	3	0	JCK-3
.....................cuggaucuuguuugacagcacucu	3	0	JCK-2
.....................cuggaucuuguuugacagcacucuc	3	0	JCK-2
.....................cuggaucuuguuugacagcacucuc	1	0	JCK-1
......................uggaucuuguuugacagA	7	1	LT-2
......................uggaucuuguuuAacagc	1	1	LCK-3
......................uggaucuugCuugacagc	1	1	JCK-2
......................uggCucuuguuugacagc	3	1	JT-1
......................uNgaucuuguuugacagc	1	1	JCK-1
......................uggaucuuguuugCcagc	1	1	JCK-3
......................ugAaucuuguuugacagc	1	1	LT-3
......................uggaucuuAuuugacagc	1	1	JCK-1
......................uggaucuuguuugacCgc	2	1	LT-1
......................ugAaucuuguuugacagc	1	1	JCK-2
......................uggaucCuguuugacagc	1	1	LT-3
......................uggaucuuguuugacCgc	2	1	JCK-2
......................uggaucuuguuugacagc	1080	0	JCK-1
......................uggaucuuguuuNacagc	3	1	LCK-1
......................uggCucuuguuugacagc	2	1	JT-3
......................uggaucuuguuugaGagc	4	1	LCK-1
......................uAgaucuuguuugacagc	4	1	LT-3
......................uggCucuuguuugacagc	53	1	LT-2
......................uNgaucuuguuugacagc	1	1	LT-3

Mature (uggaucuuguuugacagcacu) / **Star** (gugcuaucgacaugauccaga)

uccaucuuguucguuuacccucuggaucuuguuugacagcacucucuuauauuuugccauucaucuuuauaugccaaauggcgaugaugaugaugguggga gugcuaucgacaugauccagaaggugcagucgaauaagauacagagggaccacccacaguaccuacucucguuacaaucguauucuucuccucacuuauuauuauuauuauuauuauuccgaucauuucuuuuaaucuauugaag

uggaucuuguuugacagU	21	1	LT-2
uggaucuugCuugacagc	1	1	JCK-1
uggaucuuAuuugacagc	3	1	LT-1
uggCucuuguuugacagc	5	1	LT-1
uggaucuuguuugacagc	5296	0	LT-2
uggaucuuguuugacagU	13	1	LCK-3
uggaucuuguuugacagc	520	0	JT-1
uggaucuuguuugNcagc	6	1	LCK-1
uggaucuuguuugacagc	2286	0	LT-1
uggaucuuguuugCcagc	15	1	LCK-3
uggaucuuAuuugacagc	1	1	LT-3
uggaucuuguuugCcagc	3	1	JCK-1
ugNaucuuguuugacagc	7	1	LT-2
uggaucuuAuuugacagc	1	1	LCK-2
uggaucuuguuugCcagc	24	1	LT-2
uggaucuuguuugacagU	12	1	LT-3
ugUaucuuguuugacagc	1	1	JCK-1
uNgaucuuguuugacagc	1	1	LCK-3
uggaucuugCuugacagc	1	1	LT-1
uggGucuuguuugacagc	1	1	LT-2
uggaucuuguuugacagU	1	1	JCK-1
ugAaucuuguuugacagc	3	1	LCK-3
uggCucuuguuugacagc	1	1	JCK-3
uggaucuuguuugacagA	6	1	LT-3
uCgaucuuguuugacagc	1	1	LT-1
uggaucuuguuugacaCc	2	1	JCK-3
uNgaucuuguuugacagc	1	1	JT-1
uggaucuuguuugacagG	1	1	JCK-1
uAgaucuuguuugacagc	1	1	LT-1
uggaucuuguuugacCgc	1	1	JT-1
uggaucuuAuuugacagc	3	1	LT-2
uggCucuuguuugacagc	2	1	LCK-1
uggaucuuguCugacagc	2	1	JCK-2
uggaucuuguuugacGgc	1	1	LT-2
Cggaucuuguuugacagc	1	1	LT-2
ugUaucuuguuugacagc	1	1	LCK-3
uggaucuuguuNgacagc	1	1	JCK-2
uUgaucuuguuugacagc	1	1	LCK-3
uggaucuuguuugGcagc	2	1	LCK-1
uggaucuuguuNgacagc	1	1	JT-1
ugAaucuuguuugacagc	1	1	LT-1
uggaucuuguuugacagA	1	1	JCK-3
uggaucuuguuugacCgc	6	1	LT-2
uggCucuuguuugacagc	3	1	LCK-2
uggaucuuguuugacagc	1255	0	JCK-3
uggCucuuguuugacagc	2	1	JT-2
uggCucuuguuugacagc	1	1	JCK-1
uggaucuuguuugacagc	1810	0	JCK-2
uggaucuuguuugCcagc	6	1	LT-1
uggaucuuguuugacagA	4	1	LCK-3
ugUaucuuguuugacagc	1	1	LT-2
uUgaucuuguuugacagc	1	1	LT-2
uggaucuuguuugacagc	2328	0	LT-3
uggauNuuguuugacagc	1	1	LCK-1
uAgaucuuguuugacagc	5	1	LT-2
uggaucuuguuuAacagc	1	1	LCK-2
ugNaucuuguuugacagc	1	1	LCK-1
uggaucuuguuugacCgc	2	1	LCK-2
ugAaucuuguuugacagc	3	1	LT-2
uAgaucuuguuugacagc	1	1	LCK-2
uggaucuuguuugacagc	1573	0	LCK-2
uNgaucuuguuugacagc	3	1	JCK-2
uggaucuuguuuUacagc	1	1	JT-2
uggGucuuguuugacagc	1	1	LCK-3
uggaucuAguuugacagc	1	1	LT-1
uggaucuuguuugCcagc	2	1	LCK-1
uggaucuCguuugacagc	1	1	LT-1
uggCucuuguuugacagc	2	1	JCK-2
uggNucuuguuugacagc	1	1	JT-2
uggaucuuguuugaNagc	11	1	LCK-1

Mature	Star		
uccaucuuguucguuuacccucuggaucuuguuugacagcacucucuuauauuuugccauucaucuuuauaugccaaauggcgaugaugaugauggugggagugcuaucgacaugauccagaaggugcagucgaauaagauacagagggaccacccacaguaccuacucucguuacaaucguauucuucuccucacuuauuauuauuauuauuauuauuccgaucauuucuuuuaaucuauugaag			
......................uggCucuuguuugacagc...	7	1	LT-3
......................uggaucuuguuugCcagc...	4	1	LT-3
......................ugAaucuuguuugacagc...	2	1	LCK-1
......................uggaucuuguuugacagc...	6351	0	LCK-3
......................uggaucuuguuugacagA...	3	1	JCK-1
......................uggaucuuguuugacagc...	353	0	JT-2
......................uggaucuuguuugacCgc...	2	1	LCK-3
......................uggaucuuguuugacagU...	9	1	LT-1
......................uNgaucuuguuugacagc...	2	1	LCK-1
......................Cggaucuuguuugacagc...	1	1	LT-3
......................uggaucuuguuugacagc...	419	0	JT-3
......................uggaucuuguuugacagc...	1447	0	LCK-1
......................uggaucuuguuugCcagc...	4	1	JCK-2
......................uggaucuuguGugacagc...	1	1	LT-2
......................uggaucuuguuNgacagc...	1	1	JCK-1
......................uAgaucuuguuugacagc...	4	1	LCK-3
......................uUgaucuuguuugacagc...	1	1	JCK-2
......................uggaucuuguuugCcagc...	2	1	LCK-2
......................uNgaucuuguuugacagc...	1	1	JCK-3
......................uggCucuuguuugacagc...	10	1	LCK-3
......................uggNucuuguuugacagc...	1	1	JCK-1
......................uggaucuuguuugacagU...	3	1	JCK-2
......................uggaucuuguuugacagA...	1	1	LCK-1
......................uggaucuuguuugacagU...	1	1	JCK-3
......................uUgaucuuguuugacagc...	1	1	LT-3
......................uUgaucuuguuugacagca...	5	1	JT-2
......................uggCucuuguuugacagca...	4	1	JCK-2
......................uggaucuCguuugacagca...	1	1	LT-1
......................uggaucuuguuugacagUa...	18	1	LCK-3
......................uggaucuuguuugacagcG...	2	1	JT-2
......................uggCucuuguuugacagca...	2	1	LT-1
......................uggaucuuguuugacagUa...	12	1	JCK-1
......................uggaucGuguuugacagca...	1	1	LCK-2
......................uggaucuuguuugacagca...	1154	0	JCK-1
......................uggaucuuguuugacagcU...	835	1	JCK-3
......................uggaucuuguuNgacagca...	2	1	JT-1
......................uggaucuuguuugacCgca...	2	1	LCK-1
......................uNgaucuuguuugacagca...	1	1	JCK-3
......................uggGucuuguuugacagca...	1	1	LCK-2
......................uggaucuuguuugacagca...	3109	0	LCK-3
......................uggaucuuguuugacagcG...	11	1	LT-3
......................uggaucuuguuugacagcU...	1503	1	LCK-2
......................uggaucuuguuugNcagca...	9	1	LCK-1
......................uAgaucuuguuugacagca...	3	1	LCK-3
......................ugAaucuuguuugacagca...	1	1	LT-3
......................uggaucuuAuuugacagca...	1	1	LCK-3
......................uggaucuuguuugaAagca...	1	1	LCK-3
......................uggaucuuguuugCcagca...	2	1	LT-3
......................uggaucuuguuuUacagca...	1	1	LCK-3
......................uggaucuuguuugacagcU...	2784	1	LT-2
......................uggaucuuguuugCcagca...	2	1	LCK-3
......................uggaucuuguuugacagcG...	15	1	LT-2
......................uggNucuuguuugacagca...	1	1	LCK-1
......................uggaucuuguuugacCgca...	2	1	LT-3
......................uggaucuuguuugacagcC...	21	1	LCK-3
......................uggaucuuguuugacCgca...	1	1	JT-1
......................uggaucuuguuugGcagca...	1	1	LT-2
......................uggaucuuguuugacagcC...	6	1	JCK-3
......................uggaucuuguuugacagca...	1294	0	JT-3
......................uggaucuuguuugacagca...	980	0	JT-2
......................uggaucuuguuugacagcC...	7	1	LCK-1
......................uggaucuuguuugacagcG...	2	1	JCK-2
......................uggaucuuguuugacagcU...	1372	1	LCK-1
......................uggaucuuguuugacagcC...	3	1	JT-1
......................uggGucuuguuugacagca...	1	1	JCK-1
......................uggCucuuguuugacagca...	5	1	LCK-1
......................uNgaucuuguuugacagca...	1	1	LCK-3
......................uggaucuuguuugacagUa...	11	1	JT-2
......................uUgaucuuguuugacagca...	1	1	LT-3
......................uggauAuuguuugacagca...	1	1	JCK-3

```
                      Mature                                                                         Star
uccaucuuguucguuuacccucuggaucuuguuugacagcacucucuuauauuuugccauucaucuuuauaugccaaauggcgaugaugaugaugguggga gugcuaucgacaugauccagaaggugcagucgaauaagauacagagggaccacccacaguaccuacucucguuacaaucguauucuucuccucacuuauuauuauuauuauuauuauuccgaucauuuucuuuuaaucuauugaag
......................uAgaucuuguuugacagca...............................................................................................................................................................................  1      1   JT-1
......................uggaucuuguuugacagca...............................................................................................................................................................................  1862   0   JCK-2
......................uggaucuuguuugacagca...............................................................................................................................................................................  1426   0   JT-1
......................uggaucuuguuugacCgca...............................................................................................................................................................................  2      1   JCK-2
......................uggaucuuguuugacagca...............................................................................................................................................................................  1105   0   JCK-3
......................uggaucuuguuugaGagca...............................................................................................................................................................................  4      1   LCK-1
......................uggaucuuguuugacagcC..............................................................................................................................................................................  8      1   LCK-2
......................uggaCcuuguuugacagca...............................................................................................................................................................................  1      1   LT-2
......................uggaucuuguuugacagcC..............................................................................................................................................................................  2      1   JCK-1
......................uggaucuuguuugacCgca...............................................................................................................................................................................  1      1   LT-2
......................ugNaucuuguuugacagca...............................................................................................................................................................................  9      1   LT-2
......................uggaucuuguuugacagUa..............................................................................................................................................................................  11     1   LT-3
......................uggaucuuguuGgacagca...............................................................................................................................................................................  9      1   LCK-3
......................ugAaucuuguuugacagca...............................................................................................................................................................................  1      1   JCK-3
......................uggaucuuguuugaNagca...............................................................................................................................................................................  11     1   LCK-1
......................uggaucuuguuugacagca...............................................................................................................................................................................  3633   0   LCK-2
......................uggaucGuguuugacagca...............................................................................................................................................................................  1      1   JT-3
......................uggaucuugNuugacagca...............................................................................................................................................................................  1      1   LCK-1
......................uggaucuuguuugacagcU..............................................................................................................................................................................  1214   1   JCK-2
......................uggaucuuguuugCcagca...............................................................................................................................................................................  2      1   JCK-1
......................uggaucuuguuugacagca...............................................................................................................................................................................  858    0   LT-1
......................uggaucuuguuugCcagca...............................................................................................................................................................................  3      1   LCK-1
......................uggCucuuguuugacagca...............................................................................................................................................................................  2      1   LT-3
......................uggaucuugCuugacagca...............................................................................................................................................................................  1      1   JT-1
......................uggaucuuguuuNacagca...............................................................................................................................................................................  2      1   LCK-1
......................uggaucCuguuugacagca...............................................................................................................................................................................  1      1   LT-3
......................uggaucuuAuuugacagca...............................................................................................................................................................................  1      1   LCK-2
......................uggaucuuguuugacagUa..............................................................................................................................................................................  29     1   LT-2
......................uggaucuuguuugacCgca...............................................................................................................................................................................  1      1   JCK-3
......................uggaucuuguuugacagcG..............................................................................................................................................................................  6      1   JCK-1
......................uggauNuuguuugacagca...............................................................................................................................................................................  10     1   LCK-1
......................uggaucuuguuugacagcU..............................................................................................................................................................................  381    1   JT-3
......................uggaucuuguuugacagcU..............................................................................................................................................................................  279    1   JT-2
......................uggaucuuguuugacagUa..............................................................................................................................................................................  3      1   LT-1
......................uggaucuuguuugacagcC..............................................................................................................................................................................  5      1   JCK-2
......................uggaucuCguuugacagca...............................................................................................................................................................................  1      1   LT-2
......................uggauUuuguuugacagca...............................................................................................................................................................................  1      1   LT-2
......................uggaucuuguuugacCgca...............................................................................................................................................................................  2      1   JT-3
......................uggaucuuguuugacagUa..............................................................................................................................................................................  11     1   JCK-3
......................uggaucuuguuugacagcG..............................................................................................................................................................................  11     1   LCK-3
......................uAgaucuuguuugacagca...............................................................................................................................................................................  1      1   LT-2
......................uggCucuuguuugacagca...............................................................................................................................................................................  3      1   JT-2
......................uggaucuuguuNgacagca...............................................................................................................................................................................  5      1   JCK-1
......................uggaucuuguuugacagca...............................................................................................................................................................................  1490   0   LT-3
......................uggaucuuguuugacCgca...............................................................................................................................................................................  3      1   LCK-3
......................uggaucuuguuugacagcC..............................................................................................................................................................................  20     1   LT-2
......................uggaucuuguuugacagcC..............................................................................................................................................................................  14     1   LT-1
......................uggaucuuguuugacagcU..............................................................................................................................................................................  1354   1   LT-1
......................uAgaucuuguuugacagca...............................................................................................................................................................................  1      1   LT-3
......................ugUaucuuguuugacagca...............................................................................................................................................................................  1      1   LCK-2
......................uggaucuuguuugGcagca...............................................................................................................................................................................  1      1   JT-2
......................uggaucuuguuugCcagca...............................................................................................................................................................................  11     1   LT-2
......................uggaucuuguuugacagcU..............................................................................................................................................................................  2081   1   LT-3
......................uggaucuuguuugaAagca...............................................................................................................................................................................  1      1   LCK-2
......................uggaucuuguuugacagUa..............................................................................................................................................................................  10     1   JT-3
......................uggaucuuUuuugacagca...............................................................................................................................................................................  1      1   LCK-2
......................uggaucuuguuugCcagca...............................................................................................................................................................................  5      1   JCK-2
......................uggaucuuguuugacagUa..............................................................................................................................................................................  1      1   LCK-2
......................uggaucuuguuugacagcG..............................................................................................................................................................................  2      1   JCK-3
......................ugAaucuuguuugacagca...............................................................................................................................................................................  1      1   JT-1
......................uNgaucuuguuugacagca...............................................................................................................................................................................  3      1   JT-1
......................uggCucuuguuugacagca...............................................................................................................................................................................  2      1   JCK-1
......................uUgaucuuguuugacagca...............................................................................................................................................................................  5      1   JT-3
......................uggCucuuguuugacagca...............................................................................................................................................................................  4      1   LCK-3
......................uggaucCuguuugacagca...............................................................................................................................................................................  1      1   LT-2
......................uggaucuuguuugCcagca...............................................................................................................................................................................  1      1   LT-1
......................uggaucuuguuugacagcU..............................................................................................................................................................................  718    1   JCK-1
......................uggaucuCguuugacagca...............................................................................................................................................................................  1      1   LCK-2
......................uggaucuuguuugacagcU..............................................................................................................................................................................  735    1   JT-1
......................uggaucuuguuugCcagca...............................................................................................................................................................................  6      1   LCK-2
```

Mature **Star**

uccaucuuguucguuuacccucuggaucuuguuugacagcacucucuuauauuuugccauucaucuuuauaugccaaauggcgaugaugaugaugguggga gugcuaucgacaugauccagaaggugcagucgaauaagauacagagggaccacccacaguaccuacucucguuacaaucguauucuucuccucacuuauuauuauuauuauuauuauuauuccgaucauuucuuuuaaucuauugaag

......................uggaucuuguuugacagcG	12	1	LT-1
......................uggCucuuguuugacagca	5	1	JT-1
......................uggaucuuguuugacagAa	1	1	LT-3
......................uggaucuuguuugacagUa	24	1	JCK-2
......................uggCucuuguuugacagca	12	1	LT-2
......................uggaucuuguuugacagca	3565	0	LCK-1
......................ugNaucuuguuugacagca	5	1	LCK-1
......................uggaucuuguuugacagcU	4465	1	LCK-3
......................uNgaucuuguuugacagca	2	1	JCK-1
......................uggaucuuguuNgacagca	1	1	JT-2
......................uggCucuuguuugacagca	7	1	LCK-2
......................uggCucuuguuugacagca	2	1	JT-3
......................uggaucCuguuugacagca	1	1	LCK-1
......................uggaucuuguuugacCgca	1	1	JCK-1
......................uggaucuCguuugacagca	1	1	JT-1
......................uggaucuuguuugacagcC	21	1	LT-3
......................uggCucuuguuugacagca	3	1	JCK-3
......................uNgaucuuguuugacagca	2	1	JCK-2
......................uggaucuuguuugacagca	2333	0	LT-2
......................Cggaucuuguuugacagca	1	1	LCK-3
......................uAgaucuuguuugacagca	1	1	LCK-2
......................uggaucuuguuugGcagca	2	1	LCK-1
......................ugNaucuuguuugacagcac	3	1	LT-2
......................uAgaucuuguuugacagcac	1	1	LCK-1
......................uggaucuuguuugacCgcac	3	1	LT-3
......................uggaucuuguuugacCgcac	2	1	JCK-2
......................uggCucuuguuugacagcac	5	1	LCK-3
......................uggaucuuguuugacagcaU	26	1	LT-2
......................uggaucuuguuugCcagcac	1	1	JT-2
......................uggaCcuuguuugacagcac	1	1	JT-1
......................uggaucuuguuugacagcUc	51	1	LT-3
......................uggCucuuguuugacagcac	9	1	LT-2
......................uNgaucuuguuugacagcac	1	1	LCK-3
......................uggaucuuguuugaNagcac	2	1	LCK-1
......................uggaucuuguuuAacagcac	1	1	LCK-2
......................uggaucuuguuugacCgcac	1	1	LT-2
......................uggaucuuguuugacagcUc	6	1	JCK-1
......................Aggaucuuguuugacagcac	1	1	LCK-3
......................uggCucuuguuugacagcac	1	1	JCK-1
......................uNgaucuuguuugacagcac	1	1	JCK-1
......................uggCucuuguuugacagcac	1	1	LCK-2
......................uggaucuuguuugacagcaU	22	1	LT-1
......................uggaucuuguuugacagcac	249	0	JT-3
......................uggaucuuguuugacagcac	216	0	JT-1
......................uggaucuuguuugacagcaA	4	1	JCK-3
......................uggaucuuguuugacagcaU	46	1	JCK-2
......................uggaucuuguuugCcagcac	1	1	LCK-3
......................uggaucuuguuugacagcaU	16	1	JT-1
......................uggaucuuguuugacagcUc	15	1	JCK-2
......................uggaucuuCuuugacagcac	1	1	LCK-3
......................uggaucuuguuugacagcaU	26	1	JCK-3
......................uggaucuuguuugCcagcac	1	1	LT-1
......................uggaucuuguuugacagcac	586	0	JCK-1
......................uggauNuuguuugacagcac	1	1	LCK-1
......................uggaucuuguuugacagcUc	31	1	LCK-2
......................uggaucuuguuugacagcaN	2	1	JCK-1
......................uggaucuuguuugacagcUc	63	1	LCK-3
......................uggaucuuguuugCcagcac	1	1	JCK-3
......................uggaucuuguuugacagcUc	26	1	LCK-1
......................uggaucuuguuugacagcac	687	0	JCK-3
......................uggaucuuguuugCcagcac	1	1	LT-3
......................uggaucuuguuugacagcaU	8	1	JCK-1
......................ugAaucuuguuugacagcac	1	1	JCK-1
......................uggaucuuguuugacagcac	715	0	LT-3
......................uggaucuuUuuugacagcac	1	1	LT-3
......................uggaucuuguCugacagcac	1	1	JCK-2
......................uggaucuuguuugacagcCc	1	1	LT-2
......................uggaucuuguuugNcagcac	2	1	LCK-1
......................uggaucuuguuugacagcCc	1	1	JT-1
......................uggaucuuguuugacagcaA	1	1	JT-3

```
                      Mature                                                                                    Star
uccaucuuguucguuuacccucuggaucuuguuugacagcacucucuuauauuuugccauucaucuuuauaugccaaauggcgaugaugaugauggugggagugcuaucgacaugauccagaaggugcagucgaauaagauacagagggaccacccacaguaccuacucucguuacaaucguauucuucuccucacuuauuauuauuauuauuauuauuccgaucauuuucuuuuaaucuauugaag
......................uggaucuuguuugacagcac.........................................................................................................................................................................................................  1318  0  LCK-3
......................uggaucuuguuugacagcUc.........................................................................................................................................................................................................  77  1  LT-2
......................uggaucuuguuugCcagcac.........................................................................................................................................................................................................  3  1  JCK-2
......................uggaucuuguuugCcagcac.........................................................................................................................................................................................................  1  1  JCK-1
......................uggaucuuguuugacagcac.........................................................................................................................................................................................................  982  0  LT-2
......................uggaCcuuguuugacagcac.........................................................................................................................................................................................................  1  1  JCK-3
......................uggaucuuguuugacagcaA.........................................................................................................................................................................................................  3  1  LT-3
......................uAgaucuuguuugacagcac.........................................................................................................................................................................................................  4  1  LT-2
......................uggaucuuguuugacagUac.........................................................................................................................................................................................................  1  1  JCK-3
......................uggaucuuguuugGcagcac.........................................................................................................................................................................................................  1  1  LCK-1
......................uggaucuuguuugacagcUc.........................................................................................................................................................................................................  36  1  LT-1
......................uggCucuuguuugacagcac.........................................................................................................................................................................................................  1  1  LCK-1
......................uggaucuuguuugacagcNc.........................................................................................................................................................................................................  1  1  JCK-1
......................uggGucuuguuugacagcac.........................................................................................................................................................................................................  2  1  LT-2
......................uAgaucuuguuugacagcac.........................................................................................................................................................................................................  1  1  LT-1
......................uggaucuuguuugacagcUc.........................................................................................................................................................................................................  7  1  JCK-3
......................uggaucuuguuugacGgcac.........................................................................................................................................................................................................  1  1  LT-2
......................uggCucuuguuugacagcac.........................................................................................................................................................................................................  2  1  JT-3
......................uggCucuuguuugacagcac.........................................................................................................................................................................................................  2  1  JCK-2
......................uggaucuuguuugacagcac.........................................................................................................................................................................................................  899  0  JCK-2
......................uggaucuuguuugGcagcac.........................................................................................................................................................................................................  1  1  LT-3
......................uggaCcuuguuugacagcac.........................................................................................................................................................................................................  1  1  JCK-2
......................uggaucuuguuugacagcaU.........................................................................................................................................................................................................  26  1  LT-3
......................uggCucuuguuugacagcac.........................................................................................................................................................................................................  2  1  LT-3
......................uggaucuuguuugacagcGc.........................................................................................................................................................................................................  1  1  LCK-3
......................uggaucuuguuugacagcCc.........................................................................................................................................................................................................  2  1  LT-1
......................uAgaucuuguuugacagcac.........................................................................................................................................................................................................  1  1  LCK-3
......................uggaucuuguuugacagcac.........................................................................................................................................................................................................  170  0  JT-2
......................uggaucuuguuugacagcac.........................................................................................................................................................................................................  525  0  LCK-2
......................uggaucuuguuugacagcac.........................................................................................................................................................................................................  666  0  LT-1
......................uggaucuuguuugacagcaA.........................................................................................................................................................................................................  3  1  JCK-1
......................uggaucuuguuugCcagcac.........................................................................................................................................................................................................  2  1  JT-1
......................uggaucuuguuNgacagcac.........................................................................................................................................................................................................  1  1  JT-2
......................uggaucuuguuugacagcaU.........................................................................................................................................................................................................  66  1  LCK-3
......................uggaucuuguuugacagcaA.........................................................................................................................................................................................................  1  1  LCK-2
......................uggaucuuguuugacagcaG.........................................................................................................................................................................................................  1  1  LT-1
......................uggaucuuguuugCcagcac.........................................................................................................................................................................................................  4  1  LT-2
......................uggaucuuguuugacagcaA.........................................................................................................................................................................................................  1  1  JCK-2
......................uggCucuuguuugacagcac.........................................................................................................................................................................................................  1  1  LT-1
......................uggaucuuguuugacagcac.........................................................................................................................................................................................................  545  0  LCK-1
......................uggaucuugCuugacagcac.........................................................................................................................................................................................................  1  1  LT-2
......................uggaucuuguuugacaCcacu........................................................................................................................................................................................................  5  1  JCK-3
......................uggaucuuguuugacagcGcu........................................................................................................................................................................................................  3  1  LT-2
......................uggaucuuguGugacagcacu........................................................................................................................................................................................................  2  1  LT-2
......................uggaucuuguuugacaCcacu........................................................................................................................................................................................................  3  1  JCK-2
......................uggauUuuguuugacagcacu........................................................................................................................................................................................................  3  1  JCK-1
......................uggaucuuUuuugacagcacu........................................................................................................................................................................................................  3  1  JCK-1
......................Cggaucuuguuugacagcacu........................................................................................................................................................................................................  7  1  JCK-3
......................uggaucuuguuNgacagcacu........................................................................................................................................................................................................  1  1  JCK-2
......................uggaucuuguuugacagcaAu........................................................................................................................................................................................................  3  1  LT-3
......................uggaucuuguuugacagcacu........................................................................................................................................................................................................  4848  0  LCK-1
......................uggaucCuguuugacagcacu........................................................................................................................................................................................................  1  1  LCK-1
......................uggaucuuguuugacaCcacu........................................................................................................................................................................................................  5  1  JCK-1
......................uggaucuuguuugGcagcacu........................................................................................................................................................................................................  3  1  JCK-1
......................uggaucuuAuuugacagcacu........................................................................................................................................................................................................  6  1  LT-1
......................uggaucuuguuugacagcCcu........................................................................................................................................................................................................  34  1  LT-2
......................uggaCcuuguuugacagcacu........................................................................................................................................................................................................  4  1  LT-3
......................Cggaucuuguuugacagcacu........................................................................................................................................................................................................  1  1  JT-3
......................uggaucuuguuuUacagcacu........................................................................................................................................................................................................  4  1  LT-1
......................uggaucuuguuugaAagcacu........................................................................................................................................................................................................  4  1  JCK-2
......................uggaucuuguuNgacagcacu........................................................................................................................................................................................................  1  1  JT-1
......................uggaucuuguuuAacagcacu........................................................................................................................................................................................................  1  1  LT-3
......................uggaCcuuguuugacagcacu........................................................................................................................................................................................................  13  1  JCK-3
......................ugAaucuuguuugacagcacu........................................................................................................................................................................................................  2  1  LCK-1
......................uggCucuuguuugacagcacu........................................................................................................................................................................................................  9  1  LCK-2
......................ugUaucuuguuugacagcacu........................................................................................................................................................................................................  3  1  LT-3
......................uggaucuuguuugacagcacu........................................................................................................................................................................................................  1709  0  JT-1
......................uggaucuuguuugCcagcacu........................................................................................................................................................................................................  5  1  JT-1
......................uggaucuCguuugacagcacu........................................................................................................................................................................................................  10  1  JCK-2
......................uggaucuuguuugCcagcacu........................................................................................................................................................................................................  105  1  JCK-3
```

```
                      Mature                                                                         Star
uccaucuuguucguuuacccucuggaucuuguuugacagcacucucuuauauuuugccauucaucuuuauaugccaaauggcgaugaugaugauggugggagugcuaucgacaugauccagaaggugcagucgaauaagauacagagggaccacccacaguaccuacucucguuacaaucguauucuucuccucacuuauuauuauuauuauuauuauuccgaucauuuucuuuuaaucuauugaag
......................uggaucuuguuugacagAacu...............................................................................................................................................................................   3      1   LT-1
......................uggCucuuguuugacagcacu...............................................................................................................................................................................   152    1   JCK-2
......................uggaucuuguuugaAagcacu...............................................................................................................................................................................   3      1   LT-1
......................uggaucuuguuugacagGacu...............................................................................................................................................................................   24     1   JCK-1
......................uggaucuuguuugacagUacu...............................................................................................................................................................................   5      1   LT-3
......................uggaucuuguuugacGgcacu...............................................................................................................................................................................   9      1   JCK-3
......................uggaucuuAuuugacagcacu...............................................................................................................................................................................   1      1   LCK-1
......................uggaucuuguuugacagcaUu...............................................................................................................................................................................   64     1   LT-3
......................uggaucuugCuugacagcacu...............................................................................................................................................................................   6      1   JCK-3
......................uggaucuuUuuugacagcacu...............................................................................................................................................................................   6      1   JCK-3
......................uAgaucuuguuugacagcacu...............................................................................................................................................................................   5      1   JCK-1
......................uggaucuuguuugacagcacG...............................................................................................................................................................................   1      1   JT-2
......................Cggaucuuguuugacagcacu...............................................................................................................................................................................   1      1   JT-1
......................uggauUuuguuugacagcacu...............................................................................................................................................................................   11     1   JCK-2
......................uggaucGuguuugacagcacu...............................................................................................................................................................................   1      1   LT-1
......................uggCucuuguuugacagcacu...............................................................................................................................................................................   118    1   JCK-3
......................uggaucCuguuugacagcacu...............................................................................................................................................................................   10     1   JCK-1
......................uggaucuuguuugCcagcacu...............................................................................................................................................................................   36     1   LCK-3
......................uggaucuuguuugacagcacu...............................................................................................................................................................................   979    0   JT-3
......................uggaucuuguuAgacagcacu...............................................................................................................................................................................   1      1   LCK-3
......................uggaucuugCuugacagcacu...............................................................................................................................................................................   4      1   LT-2
......................uggaucuugGuugacagcacu...............................................................................................................................................................................   1      1   JCK-3
......................uggaucuuAuuugacagcacu...............................................................................................................................................................................   3      1   LT-3
......................uggauNuuguuugacagcacu...............................................................................................................................................................................   3      1   LCK-1
......................uggaucuuguuugacagcaAu...............................................................................................................................................................................   10     1   JCK-2
......................uggaucuuguuugacagcCcu...............................................................................................................................................................................   4      1   LCK-1
......................uggaucuuguuugacagcUcu...............................................................................................................................................................................   1      1   LT-3
......................uggaucuuguuugacagcaUu...............................................................................................................................................................................   37     1   LCK-2
......................uNgaucuuguuugacagcacu...............................................................................................................................................................................   104    1   JCK-2
......................uggaucuuguuugacagcCcu...............................................................................................................................................................................   31     1   LT-3
......................uAgaucuuguuugacagcacu...............................................................................................................................................................................   30     1   LT-2
......................uggaucuuguuugacGgcacu...............................................................................................................................................................................   10     1   JCK-2
......................uggaucuuguuugacagcaAu...............................................................................................................................................................................   4      1   LT-2
......................uggaucuuguuGgacagcacu...............................................................................................................................................................................   10     1   JCK-1
......................uggaucuuCuuugacagcacu...............................................................................................................................................................................   1      1   JCK-2
......................uggaucuuguuugaGagcacu...............................................................................................................................................................................   8      1   LCK-1
......................ugCaucuuguuugacagcacu...............................................................................................................................................................................   1      1   JCK-3
......................uggaucuGguuugacagcacu...............................................................................................................................................................................   1      1   JCK-2
......................uggaucuuguuugacCgcacu...............................................................................................................................................................................   28     1   JCK-3
......................uggaucuuguuugacagcUcu...............................................................................................................................................................................   5      1   LT-1
......................uggaucuuguuugacagcGcu...............................................................................................................................................................................   2      1   JCK-1
......................uggaucuuUuuugacagcacu...............................................................................................................................................................................   2      1   LT-1
......................ugAaucuuguuugacagcacu...............................................................................................................................................................................   3      1   JCK-1
......................ugAaucuuguuugacagcacu...............................................................................................................................................................................   19     1   JCK-2
......................uggaucuuguuugacagcaUu...............................................................................................................................................................................   36     1   LCK-1
......................ugAaucuuguuugacagcacu...............................................................................................................................................................................   4      1   LCK-3
......................uAgaucuuguuugacagcacu...............................................................................................................................................................................   15     1   JCK-3
......................uNgaucuuguuugacagcacu...............................................................................................................................................................................   3      1   LT-1
......................uggaucuuguuugacagcacu...............................................................................................................................................................................   23834  0   LT-1
......................uggaucuuguuugacagcCcu...............................................................................................................................................................................   16     1   LCK-3
......................Cggaucuuguuugacagcacu...............................................................................................................................................................................   6      1   JCK-1
......................uggaucuuUuuugacagcacu...............................................................................................................................................................................   1      1   LCK-3
......................uggaucuugGuugacagcacu...............................................................................................................................................................................   1      1   JCK-1
......................uggauAuuguuugacagcacu...............................................................................................................................................................................   2      1   JCK-2
......................Cggaucuuguuugacagcacu...............................................................................................................................................................................   4      1   LT-2
......................uggaucuuCuuugacagcacu...............................................................................................................................................................................   1      1   JCK-3
......................uUgaucuuguuugacagcacu...............................................................................................................................................................................   10     1   JCK-3
......................ugUaucuuguuugacagcacu...............................................................................................................................................................................   2      1   LT-2
......................uggaucuugCuugacagcacu...............................................................................................................................................................................   6      1   JCK-2
......................uggaucuuguuugacagcaNu...............................................................................................................................................................................   42     1   JCK-1
......................uggaucuuguCugacagcacu...............................................................................................................................................................................   7      1   JCK-3
......................uggaucuuguuugCcagcacu...............................................................................................................................................................................   5      1   LCK-1
......................uggaucuuguuugacGgcacu...............................................................................................................................................................................   2      1   LCK-3
......................uggaucuuguuAgacagcacu...............................................................................................................................................................................   1      1   JCK-1
......................uggaucuuguuuAacagcacu...............................................................................................................................................................................   1      1   LCK-3
......................uggauUuuguuugacagcacu...............................................................................................................................................................................   4      1   JCK-3
......................uNgaucuuguuugacagcacu...............................................................................................................................................................................   66     1   JCK-1
......................uggaucuuguuugacagcacu...............................................................................................................................................................................   27651  0   LT-3
......................uggaucuuguuugacGgcacu...............................................................................................................................................................................   1      1   JT-3
......................uggaucuuguuugacagcCcu...............................................................................................................................................................................   29     1   LT-1
```

Mature **Star**

uccaucuuguucguuuacccucuggaucuuguuugacagcacucucuuauauuuugccauucaucuuuauaugccaaauggcgaugaugaugauggugggagugcuaucgacaugauccagaaggugcagucgaauaagauacagagggaccacccacaguaccuacucucguuacaaucguauucuucuccucacuuauuauuauuauuauuauuauuccgaucauuuucuuuuaaucuauugaag

uggaucuuguuugacagcaUu	68	1	LT-2
uNgaucuuguuugacagcacu	4	1	JT-1
uggaucuuguCugacagcacu	5	1	JT-2
uggaucuuguuugaAagcacu	5	1	JCK-3
uggaucuuguuugacGgcacu	2	1	LT-1
uggaucuuUuuugacagcacu	1	1	LT-3
uggaucuuguuugacCgcacu	3	1	JT-1
uggaucuuguuugacagcacu	20839	0	LT-2
Cggaucuuguuugacagcacu	9	1	LT-3
uggaucuuguuugacagcacG	10	1	LT-1
uggaucuuguuugacagcCcu	74	1	JCK-2
uggaucuuguuugacagcGcu	1	1	JT-3
uggaucuuguuugacagcaUu	21	1	JT-3
uggaucuuguCugacagcacu	1	1	LT-3
uggauAuuguuugacagcacu	1	1	JCK-3
uggaucuuguuugacagcacN	22	1	JCK-1
uggaucuuguuugacGgcacu	7	1	JCK-1
uggaCcuuguuugacagcacu	3	1	LT-1
uggaucuugCuugacagcacu	1	1	LT-3
uggaucuuguuugGcagcacu	2	1	LT-1
uggaucuuguuuUacagcacu	2	1	JCK-3
uggNucuuguuugacagcacu	1	1	LCK-1
uggaucuuguuuUacagcacu	2	1	LT-2
uggaucuuguuugGcagcacu	1	1	LT-3
uggaucuuguuugacagcaUu	20	1	LT-1
uggaucuuCuuugacagcacu	1	1	JCK-1
uggaucuuguuugacagcaAu	3	1	JCK-3
uggaucuugAuugacagcacu	1	1	JCK-2
uggaucuuUuuugacagcacu	3	1	LT-2
uggGucuuguuugacagcacu	3	1	LT-3
uggauUuuguuugacagcacu	2	1	LT-3
ugNaucuuguuugacagcacu	65	1	LT-2
uggaucuuguuugacagUacu	1	1	JT-2
uggaucuuguuugacagUacu	18	1	JCK-1
uggaucuuguuuCacagcacu	1	1	JCK-2
uggauNuuguuugacagcacu	1	1	JCK-2
uggaucuuguCugacagcacu	3	1	LT-1
uggGucuuguuugacagcacu	8	1	JCK-1
uggaucuuguuGgacagcacu	1	1	JCK-2
uAgaucuuguuugacagcacu	14	1	LT-1
uggNucuuguuugacagcacu	1	1	JCK-2
uggaucuugCuugacagcacu	2	1	LT-1
ugAaucuuguuugacagcacu	11	1	LT-3
uggaucuuguuugacagcacu	658	0	JT-2
uggaCcuuguuugacagcacu	1	1	LCK-2
uAgaucuuguuugacagcacu	8	1	JCK-2
uggaucuuguuugacagcaUu	1426	1	JCK-1
uggaucuuguuugacagcacu	5029	0	LCK-2
uggaucCuguuugacagcacu	2	1	LT-2
uggNucuuguuugacagcacu	3	1	JCK-3
uggaucuuguuugacagcacG	16	1	JCK-2
uggaucuuAuuugacagcacu	1	1	LCK-2
uggaucuuguuugacagcaGu	2	1	JCK-1
uggaucuuguuugacagcaAu	1	1	LCK-2
uggaucCuguuugacagcacu	12	1	JCK-2
uggaucuuguuugGcagcacu	3	1	JCK-3
uggCucuuguuugacagcacu	65	1	LT-1
ugUaucuuguuugacagcacu	2	1	JCK-1
uggaucuCguuugacagcacu	5	1	JCK-1
uAgaucuuguuugacagcacu	22	1	LT-3
uggaucuuguuugacagcacu	20263	0	LCK-3
uggaucCuguuugacagcacu	2	1	LT-3
uggaucuuguuugacagcaAu	4	1	JT-3
uggaucuuguuugacagcGcu	2	1	JCK-2
ugCaucuuguuugacagcacu	1	1	LCK-3
uggaucuuguuuUacagcacu	3	1	LT-3
uUgaucuuguuugacagcacu	2	1	LCK-3
uggaucuuguCugacagcacu	1	1	LCK-2
uNgaucuuguuugacagcacu	1	1	LCK-2
uggaucuuguuugacCgcacu	5	1	LCK-2

Mature **Star**

uccaucuuguucguuuacccucuggaucuuguuugacagcacucucuuauauuuugccauucaucuuuauaugccaaauggcgaugaugaugauggugga gugcuaucgacaugauccagaaggugcagucgaauaagauacagagggaccacccacaguaccuacucucguuacaaucguauucuucuccucacuuauuauuauuauuauuauuauuccgaucauuucuuuuaaucuauugaag

uggauGuuguuugacagcacu	1	1	JCK-3
uggaucuuguuugacagcNcu	1	1	LCK-1
uggaucuuguuugacCgcacu	12	1	LT-1
uggaucuCguuugacagcacu	4	1	JCK-3
uggaucuuguuuAacagcacu	3	1	LT-1
uggaucuuguuugacagcaUu	55	1	JT-1
uggaucuuguuugacagcUcu	1	1	JCK-2
uggaucuuguuugCcagcacu	159	1	JCK-2
uggaucuuguuugacagcacG	10	1	JCK-1
uggaucuuguuugacGgcacu	7	1	LT-2
uggaucuuguuugacagcaUu	22	1	JT-2
uggaucuCguuugacagcacu	1	1	LT-1
uUgaucuuguuugacagcacu	7	1	LT-2
uggaucuCguuugacagcacu	3	1	LT-3
uggNucuuguuugacagcacu	22	1	JCK-1
ugAaucuuguuugacagcacu	3	1	LCK-2
uggGucuuguuugacagcacu	8	1	JCK-2
uggCucuuguuugacagcacu	1	1	JT-3
uggaucuuUuuugacagcacu	4	1	JCK-2
uggaucuugCuugacagcacu	2	1	JCK-1
uggaucuuguuugacagGacu	2	1	JCK-2
uggaucuuguuugacagcaUu	1732	1	JCK-3
uggaucuuguuuAacagcacu	6	1	JCK-1
uggaucuuguuugacagcacu	48441	0	JCK-3
uNgaucuuguuugacagcacu	2	1	LT-3
uggaucuuguuugCcagcacu	2	1	JT-2
uggaucuuguuuUacagcacu	6	1	JCK-2
uggaucuuguuugacaCcacu	1	1	LT-3
uggaucCuguuugacagcacu	8	1	JCK-3
Gggaucuuguuugacagcacu	1	1	JCK-2
uggCucuuguuugacagcacu	95	1	LT-3
uggaGcuuguuugacagcacu	2	1	JCK-3
Cggaucuuguuugacagcacu	11	1	JCK-2
uggaucuuguuugacagUacu	13	1	JCK-3
uggaucuuguuugCcagcacu	1	1	JT-3
uggaucuuguuugacCgcacu	3	1	LCK-1
Cggaucuuguuugacagcacu	3	1	LT-1
uggCucuuguuugacagcacu	92	1	JCK-1
uUgaucuuguuugacagcacu	1	1	JT-3
uggaucuuguuugacagcNcu	22	1	JCK-1
uggCucuuguuugacagcacu	2	1	JT-2
uggaucuuguuugCcagcacu	7	1	LCK-2
uggaucuuguuugaGagcacu	1	1	JT-1
uggaucuuguuugacCgcacu	26	1	LT-2
ugUaucuuguuugacagcacu	1	1	LCK-2
uggauUuuguuugacagcacu	1	1	LCK-3
uggaucuuguuugacagcacG	7	1	LCK-3
uggCucuuguuugacagcacu	7	1	JT-1
uggCucuuguuugacagcacu	8	1	LCK-1
ugAaucuuguuugacagcacu	6	1	LT-1
uggaucuuguCugacagcacu	1	1	LT-2
uggaucuuAuuugacagcacu	4	1	LCK-3
uggaCcuuguuugacagcacu	4	1	JCK-1
uggaucuuguuugacagcaAu	1	1	LCK-3
uggaucuuguuugacagUacu	34	1	JCK-2
uggaucuuguuugacGgcacu	3	1	LT-3
uggaucuugGuugacagcacu	1	1	JCK-2
uggaucuuguuugacCgcacu	7	1	LCK-3
uggaucuuguuugacagcaAu	1	1	LT-1
uggaucuuguuuUacagcacu	6	1	JCK-1
uggauUuuguuugacagcacu	1	1	LT-2
uggaucuuguuugacagcacu	66730	0	JCK-2
uggaucuuguuugCcagcacu	67	1	LT-3
uggaucuuguuugacagAacu	1	1	JCK-1
uggaucuuAuuugacagcacu	20	1	JCK-2
uggaucuuguuugacagcacG	12	1	LT-3
uggCucuuguuugacagcacu	39	1	LCK-3
ugUaucuuguuugacagcacu	1	1	JCK-2
uggaCcuuguuugacagcacu	2	1	LT-2
uggaucuuguuugacaCcacu	1	1	JT-1

```
                      Mature                                                                                    Star
uccaucuuguucguuuacccucuggaucuuguuugacagcacucucuuauauuuugccauucaucuuuauaugccaaauggcgaugaugaugaugguggagugcuaucgacaugauccagaaggugcagucgaauaagauacagagggaccacccacaguaccuacucucguuacaaucguauucuucuccucacuuauuauuauuauuauuauuauuccgaucauuuucuuuuaaucuauugaag
......................ugNaucuuguuugacagcacu............................................................................................................................................................................................    1      1     LCK-1
......................uggaucuuguuugGcagcacu............................................................................................................................................................................................    3      1     JCK-2
......................uggaucuuguCugacagcacu............................................................................................................................................................................................    6      1     JCK-1
......................uggaucuuguCugacagcacu............................................................................................................................................................................................    6      1     JCK-2
......................uggaucuuguuugacagcaAu............................................................................................................................................................................................    5      1     JT-2
......................uggaucuuguuugCcagcacu............................................................................................................................................................................................    61     1     LT-1
......................uggaucuugCuugacagcacu............................................................................................................................................................................................    4      1     LCK-3
......................uggaucGuguuugacagcacu............................................................................................................................................................................................    2      1     JCK-1
......................uggaucuuguuuAacagcacu............................................................................................................................................................................................    1      1     LCK-1
......................uggaucuuguuugacagGacu............................................................................................................................................................................................    2      1     JCK-3
......................ugUaucuuguuugacagcacu............................................................................................................................................................................................    1      1     JT-1
......................ugAaucuuguuugacagcacu............................................................................................................................................................................................    8      1     JCK-3
......................uggaucCuguuugacagcacu............................................................................................................................................................................................    4      1     LT-1
......................uggaucuuguuugacagcacu............................................................................................................................................................................................    38814  0     JCK-1
......................uggaucuuguuugacagcCcu............................................................................................................................................................................................    17     1     JCK-3
......................uggaucuuguuugaAagcacu............................................................................................................................................................................................    2      1     JCK-1
......................uggaucuuguuugacagcCcu............................................................................................................................................................................................    4      1     LCK-2
......................uggCucuuguuugacagcacu............................................................................................................................................................................................    200    1     LT-2
......................uggaucuuguuuAacagcacu............................................................................................................................................................................................    9      1     JCK-2
......................uggGucuuguuugacagcacu............................................................................................................................................................................................    5      1     JCK-3
......................uNgaucuuguuugacagcacu............................................................................................................................................................................................    55     1     JCK-3
......................uggaucuuguuugacagNacu............................................................................................................................................................................................    3      1     JCK-3
......................uggaucuCguuugacagcacu............................................................................................................................................................................................    1      1     LCK-1
......................uggaucuuguuGgacagcacu............................................................................................................................................................................................    1      1     LT-3
......................uggaucuuAuuugacagcacu............................................................................................................................................................................................    9      1     LT-2
......................uggaucuuguuugacCgcacu............................................................................................................................................................................................    29     1     JCK-2
......................uggaCcuuguuugacagcacu............................................................................................................................................................................................    1      1     LCK-3
......................uggaucuuguuugUcagcacu............................................................................................................................................................................................    2      1     JCK-2
......................uggaucuuguuugacagcaAu............................................................................................................................................................................................    3      1     JCK-1
......................uUgaucuuguuugacagcacu............................................................................................................................................................................................    4      1     JCK-2
......................uggaucuuAuuugacagcacu............................................................................................................................................................................................    6      1     JCK-1
......................uggaucuuguuugacCgcacu............................................................................................................................................................................................    19     1     JCK-1
......................uggaucuuguuugacagcaUu............................................................................................................................................................................................    2361   1     JCK-2
......................uggaucuuguGugacagcacu............................................................................................................................................................................................    15     1     JCK-1
......................uggaucuuguuugacagUacu............................................................................................................................................................................................    11     1     LT-2
......................uggaCcuuguuugacagcacu............................................................................................................................................................................................    14     1     JCK-2
......................uggaucuuguuugacagcCcu............................................................................................................................................................................................    2      1     JT-1
......................ugUaucuuguuugacagcacu............................................................................................................................................................................................    4      1     JCK-3
......................uggaucuuguuuAacagcacu............................................................................................................................................................................................    2      1     LT-2
......................uNgaucuuguuugacagcacu............................................................................................................................................................................................    1      1     LCK-1
......................uggaucuuguuugacagcacG............................................................................................................................................................................................    13     1     JCK-3
......................uggaucuuguuugCcagcacu............................................................................................................................................................................................    86     1     JCK-1
......................ugAaucuuguuugacagcacu............................................................................................................................................................................................    10     1     LT-2
......................uggaucuuguuugCcagcacu............................................................................................................................................................................................    80     1     LT-2
......................uggaucuuguuAgacagcacu............................................................................................................................................................................................    1      1     JCK-2
......................uggaucuuguuugGcagcacu............................................................................................................................................................................................    1      1     LT-2
......................uCgaucuuguuugacagcacu............................................................................................................................................................................................    1      1     JCK-3
......................uggGucuuguuugacagcacu............................................................................................................................................................................................    1      1     JT-1
......................uggaucuuguuuAacagcacu............................................................................................................................................................................................    1      1     JT-1
......................uggaucuuguuugacagcCcu............................................................................................................................................................................................    53     1     JCK-1
......................uggaucuuguuGgacagcacu............................................................................................................................................................................................    1      1     JCK-3
......................uggaucuuguuugacagcacG............................................................................................................................................................................................    3      1     LT-2
......................uggaucuuguuuNacagcacu............................................................................................................................................................................................    1      1     LCK-1
......................uggaucuuguuugacagcaUu............................................................................................................................................................................................    102    1     LCK-3
......................uAgaucuuguuugacagcacu............................................................................................................................................................................................    4      1     LCK-2
......................uggaucuuguuugacagcacG............................................................................................................................................................................................    2      1     LCK-1
......................uggaucuuguuugacagcUcu............................................................................................................................................................................................    6      1     LT-2
......................uggaucuuNuuugacagcacu............................................................................................................................................................................................    1      1     JCK-3
......................uggaucuuguuuAacagcacu............................................................................................................................................................................................    7      1     JCK-3
......................uUgaucuuguuugacagcacu............................................................................................................................................................................................    2      1     LT-1
......................uggaucuuguuugacagAacu............................................................................................................................................................................................    1      1     JCK-2
......................uAgaucuuguuugacagcacu............................................................................................................................................................................................    13     1     LCK-3
......................ugCaucuuguuugacagcacu............................................................................................................................................................................................    2      1     LT-2
......................uggaucuuguuuCacagcacu............................................................................................................................................................................................    1      1     JCK-3
......................uggaucuuAuuugacagcacu............................................................................................................................................................................................    4      1     JCK-3
......................uggaucuuguuugNcagcacu............................................................................................................................................................................................    15     1     LCK-1
......................uCgaucuuguuugacagcacu............................................................................................................................................................................................    1      1     LCK-3
......................uggaucuuguuugGcagcacu............................................................................................................................................................................................    5      1     LCK-1
......................uggaucuuguuugaNagcacu............................................................................................................................................................................................    17     1     LCK-1
......................uggaucuuguuugacCgcacu............................................................................................................................................................................................    11     1     LT-3
```

Mature (uggaucuuguuugacagcacu) … **Star** (gugcuaucgacaugauccaga)

uccaucuuguucguuuacccucuggaucuuguuugacagcacucucuuauauuuugccauucaucuuuauaugccaaauggcgaugaugaugaugguggagugcuaucgacaugauccagaaggugcagucgaauaagauacagagggaccacccacaguaccuacucucguuacaaucguauucuucuccucacuuauuauuauuauuauuauuauuccgaucauuucuuuuaaucuauugaag

uggaucuuguuuCacagcacu	2	1	JCK-1
uggaucuuguuNgacagcacu	121	1	JCK-1
uggaucuCguuugacagcacu	3	1	LCK-3
uggGucuuguuugacagcacuc	4	1	JCK-2
uggaucuuguuugacagcCcuc	7	1	JCK-2
uggaucuuguuugCcagcacuc	2	1	JT-1
uggaucuuguuugacagcacuA	33	1	LT-2
uAgaucuuguuugacagcacuc	2	1	LT-1
uggCucuuguuugacagcacuc	4	1	LT-1
uggaucuuguuugacaAcacuc	6	1	JCK-3
ugCaucuuguuugacagcacuc	1	1	JCK-2
uggaucuuguuNgacagcacuc	29	1	JCK-1
uggaucuuguuugCcagcacuc	19	1	JCK-3
uggCucuuguuugacagcacuc	7	1	LT-3
uggaucuuguuugacagcacuc	1069	0	LT-3
uggaucuuguuugacagcacuU	1	1	JT-2
uggaucuuguuugacagcacuc	70	0	LCK-2
uggaucuuguuugacagcaUuc	8	1	JCK-3
uNgaucuuguuugacagcacuc	17	1	JCK-2
uggaucuuguuugacagcacuU	47	1	LT-1
uggaucuuguuugacagNacuc	1	1	JCK-3
uggaucuuguuugacagcacuU	22	1	LCK-2
uggaucuuguuugacagcacuc	76	0	LCK-1
uggaucuuguuugacGgcacuc	2	1	JCK-3
uggaucuuguuugacagcacGc	1	1	JCK-1
uggaucuugCuugacagcacuc	1	1	JCK-2
uUgaucuuguuugacagcacuc	1	1	JCK-2
uggaucuugGuugacagcacuc	3	1	JCK-2
uggaucuuAuuugacagcacuc	4	1	JCK-2
uggaucuuguuugacGgcacuc	1	1	LT-1
ugUaucuuguuugacagcacuc	1	1	JCK-2
uggaucuuguuugacagcacuU	18	1	LCK-1
uggCucuuguuugacagcacuc	2	1	LCK-1
uggaucuuguuugacagcacuG	11	1	JCK-1
uggaucuuguuugCcagcacuc	24	1	JCK-1
uggaucuuguuugacagcUcuc	2	1	JCK-2
ugNaucuuguuugacagcacuc	2	1	LT-2
uAgaucuuguuugacagcacuc	7	1	JCK-2
uggaucuCguuugacagcacuc	2	1	JCK-1
ugAaucuuguuugacagcacuc	1	1	LT-1
uggaucuuguuugacagcacuG	3	1	LT-3
uggaucuuAuuugacagcacuc	4	1	JCK-3
uggaucuuguuugacagcacuA	41	1	JCK-3
uggaucuuguuugGcagcacuc	3	1	JCK-2
uggaucuuguuugacagcacuA	12	1	LCK-3
ugAaucuuguuugacagcacuc	2	1	JCK-3
uggaucuuguuugacagcacGc	1	1	JCK-2
uggaucuuguuugacagcacuA	11	1	JT-1
uggaucuuguuugacagcacuU	68	1	JCK-1
ugUaucuuguuugacagcacuc	1	1	JCK-1
uAgaucuuguuugacagcacuc	1	1	LT-3
ugAaucuuguuugacagcacuc	1	1	LT-2
uggaucuuguuugaGagcacuc	4	1	JCK-2
uggaucuuUuuugacagcacuc	1	1	JCK-3
uggauUuuguuugacagcacuc	1	1	JCK-3
uggaucCuguuugacagcacuc	1	1	JCK-1
uggaucuuguuugacagcNcuc	7	1	JCK-1
uggaucuuguuugacagcacuc	152	0	JT-3
uggaucuuguuugacagUacuc	5	1	JCK-2
uggaucuuguuugCcagcacuc	1	1	JT-2
uggauUuuguuugacagcacuc	5	1	JCK-1
uggaucuCguuugacagcacuc	3	1	JCK-2
uggaucuuguuugacCgcacuc	1	1	LT-2
uggaucuuguuugacagcCcuc	7	1	JCK-1
uggaucuuguuugCcagcacuc	2	1	LT-2
uUgaucuuguuugacagcacuc	1	1	JCK-1
ugAaucuuguuugacagcacuc	2	1	JCK-1
uggaucuuguuugacagcaNuc	11	1	JCK-1
uggaucuuguuugCcagcacuc	2	1	LT-3
uggaucuuguuugacagcacuA	59	1	JCK-2

```
                      Mature                                                                         Star
uccaucuuguucguuuacccucuggaucuuguuugacagcacucucuuauauuuugccauucaucuuuauaugccaaauggcgaugaugaugaugguggagugcuaucgacaugauccagaaggugcagucgaauaagauacagagggaccacccacaguaccuacucucguuacaaucguauucuucuccucacuuauuauuauuauuauuauuauuccgaucauuuucuuuuaaucuauugaag
......................uggaucuuguuugacagcaUuc..............................................................................................................................................................................................................   5   1   JCK-2
......................uggCucuuguuugacagcacuc..............................................................................................................................................................................................................   3   1   LCK-3
......................uggaucuuguuGgacagcacuc..............................................................................................................................................................................................................   3   1   JCK-1
......................uggaucuuAuuugacagcacuc..............................................................................................................................................................................................................   1   1   LT-2
......................uggCucuuguuugacagcacuc..............................................................................................................................................................................................................   33   1   JCK-3
......................uAgaucuuguuugacagcacuc..............................................................................................................................................................................................................   3   1   JCK-1
......................uggaucuuguuuAacagcacuc..............................................................................................................................................................................................................   3   1   JCK-2
......................Cggaucuuguuugacagcacuc..............................................................................................................................................................................................................   6   1   JCK-2
......................uggaucuuguuugacagcacuN..............................................................................................................................................................................................................   4   1   LT-2
......................uggCucuuguuugacagcacuc..............................................................................................................................................................................................................   36   1   JCK-2
......................uggaucuuguuugacagcCcuc..............................................................................................................................................................................................................   1   1   JT-3
......................uggaGcuuguuugacagcacuc..............................................................................................................................................................................................................   1   1   JCK-3
......................uggaucuuguuugacCgcacuc..............................................................................................................................................................................................................   2   1   LCK-3
......................uggaucuuguuugacagcacuU..............................................................................................................................................................................................................   48   1   LT-3
......................uggaucuuguuugacCgcacuc..............................................................................................................................................................................................................   3   1   JCK-3
......................uggaucuuguuugacCgcacuc..............................................................................................................................................................................................................   5   1   JCK-1
......................uggGucuuguuugacagcacuc..............................................................................................................................................................................................................   1   1   JCK-1
......................uggaucuuguuugacCgcacuc..............................................................................................................................................................................................................   1   1   LT-1
......................uggNucuuguuugacagcacuc..............................................................................................................................................................................................................   7   1   JCK-1
......................uggCucuuguuugacagcacuc..............................................................................................................................................................................................................   4   1   LT-2
......................ugUaucuuguuugacagcacuc..............................................................................................................................................................................................................   3   1   JCK-3
......................uggGucuuguuugacagcacuc..............................................................................................................................................................................................................   1   1   JCK-3
......................uggaucuuguuugacagcacuc..............................................................................................................................................................................................................   9941   0   JCK-1
......................uggaucuuguuugacagcacuU..............................................................................................................................................................................................................   64   1   LCK-3
......................uNgaucuuguuugacagcacuc..............................................................................................................................................................................................................   26   1   JCK-1
......................Cggaucuuguuugacagcacuc..............................................................................................................................................................................................................   5   1   JCK-3
......................uggaucuuguuugacagcacuU..............................................................................................................................................................................................................   85   1   JCK-3
......................uggaucuuguuugacagcacuc..............................................................................................................................................................................................................   17179   0   JCK-2
......................uggaucuuguuugCcagcacuc..............................................................................................................................................................................................................   3   1   LCK-3
......................ugAaucuuguuugacagcacuc..............................................................................................................................................................................................................   2   1   LT-3
......................uggaucuuguuugacagcacuA..............................................................................................................................................................................................................   38   1   JCK-1
......................uAgaucuuguuugacagcacuc..............................................................................................................................................................................................................   10   1   JCK-3
......................uggaucuuguuugacaAcacuc..............................................................................................................................................................................................................   3   1   JCK-2
......................uggaucCuguuugacagcacuc..............................................................................................................................................................................................................   2   1   JCK-2
......................uggaucuuguuugNcagcacuc..............................................................................................................................................................................................................   1   1   LCK-1
......................uNgaucuuguuugacagcacuc..............................................................................................................................................................................................................   15   1   JCK-3
......................uggaucuuguuugacagcUcuc..............................................................................................................................................................................................................   1   1   JCK-1
......................uggaucuuguuugacagcaUuc..............................................................................................................................................................................................................   3   1   LT-2
......................uggaucuuguGugacagcacuc..............................................................................................................................................................................................................   5   1   JCK-1
......................uUgaucuuguuugacagcacuc..............................................................................................................................................................................................................   1   1   LT-2
......................uggaCcuuguuugacagcacuc..............................................................................................................................................................................................................   9   1   JCK-3
......................uggaucuuguuugacagcacNc..............................................................................................................................................................................................................   3   1   JCK-1
......................uggCucuuguuugacagcacuc..............................................................................................................................................................................................................   36   1   JCK-1
......................uggaucuuguuugacagcCcuc..............................................................................................................................................................................................................   1   1   LCK-1
......................uUgaucuuguuugacagcacuc..............................................................................................................................................................................................................   1   1   JCK-3
......................uggaucuuguuugacagcacuN..............................................................................................................................................................................................................   9   1   JCK-1
......................uggaCcuuguuugacagcacuc..............................................................................................................................................................................................................   1   1   LT-2
......................uAgaucuuguuugacagcacuc..............................................................................................................................................................................................................   1   1   LT-2
......................uggaucuuguuugacagcacuc..............................................................................................................................................................................................................   12872   0   JCK-3
......................uggaucuuguuugacagcacuU..............................................................................................................................................................................................................   97   1   JCK-2
......................uggaCcuuguuugacagcacuc..............................................................................................................................................................................................................   6   1   JCK-1
......................uggaucuugGuugacagcacuc..............................................................................................................................................................................................................   3   1   JCK-1
......................uggauUuuguuugacagcacuc..............................................................................................................................................................................................................   7   1   JCK-2
......................uggaucuuguuugacagcacuc..............................................................................................................................................................................................................   520   0   LCK-3
......................uggaucuuNuuugacagcacuc..............................................................................................................................................................................................................   1   1   JCK-3
......................uggaucCuguuugacagcacuc..............................................................................................................................................................................................................   5   1   JCK-3
......................uggaucuuguuugacagcacuc..............................................................................................................................................................................................................   138   0   JT-2
......................uggaucuuguuugacagUacuc..............................................................................................................................................................................................................   1   1   JCK-1
......................uggaucuuguuugacagcacuG..............................................................................................................................................................................................................   1   1   LT-2
......................uggaucuuguuugacagGacuc..............................................................................................................................................................................................................   7   1   JCK-1
......................uggaucuuguuugCcagcacuc..............................................................................................................................................................................................................   44   1   JCK-2
......................uggCucuuguuugacagcacuc..............................................................................................................................................................................................................   1   1   JT-1
......................uggaucuuguuugacagcCcuc..............................................................................................................................................................................................................   9   1   JCK-3
......................uggaucuuguuugacagcacuG..............................................................................................................................................................................................................   7   1   LT-1
......................Cggaucuuguuugacagcacuc..............................................................................................................................................................................................................   2   1   JCK-1
......................uggaucuuguuuAacagcacuc..............................................................................................................................................................................................................   1   1   JCK-3
......................uggaucuuguuugacagcacuG..............................................................................................................................................................................................................   4   1   JCK-2
......................uggaucuCguuugacagcacuc..............................................................................................................................................................................................................   2   1   JCK-3
......................uggaucuuguuugacagcacuA..............................................................................................................................................................................................................   34   1   LT-3
......................uAgaucuuguuugacagcacuc..............................................................................................................................................................................................................   1   1   LCK-3
```

Mature **Star**

uccaucuuguucguuuacccucuggaucuuguuugacagcacucucuuauauuuugccauucaucuuuauaugccaaauggcgaugaugaugaugguggga gugcuaucgacaugauccagaaggugcagucgaauaagauacagagggaccacccacaguaccuacucucguuacaaucguauucuucuccucacuuauuauuauuauuauuauuauuccgaucauuuucuuuuaaucuauugaag			
......................uggaucuuguCugacagcacuc	1	1	JCK-1
......................uggaucuuguuugacagcacuU	4	1	JT-3
......................uggCucuuguuugacagcacuc	1	1	JT-2
......................uggaucuuguuugacaAcacuc	4	1	JCK-1
......................uggaucuugGuugacagcacuc	3	1	JCK-3
......................uggaucuugCuugacagcacuc	1	1	JCK-3
......................uggaucuuguuugacagcaUuc	7	1	JCK-1
......................uggaucuuguuugGcagcacuc	1	1	JCK-1
......................ugAaucuuguuugacagcacuc	4	1	JCK-2
......................Cggaucuuguuugacagcacuc	1	1	LCK-3
......................uggaucuuguuugacagcacuc	627	0	LT-2
......................uggaucuuguuugCcagcacuc	7	1	LT-1
......................uggauAuuguuugacagcacuc	1	1	JCK-1
......................uggaucuuguuuUacagcacuc	1	1	JCK-1
......................uggaucuuguuugacagcacuc	1262	0	LT-1
......................uggaucuuguuugaAagcacuc	4	1	JCK-2
......................uggaucuuUuuugacagcacuc	2	1	JCK-2
......................uggaucuuguuugacCgcacuc	13	1	JCK-2
......................Cggaucuuguuugacagcacuc	1	1	LT-2
......................uggaucuuguuugacagcacuc	220	0	JT-1
......................uggaucuuguuugacagcacuA	32	1	LT-1
......................uggaucuuguuugacagcacGc	1	1	JCK-3
......................uggaucuuguuugacagcacuG	2	1	JCK-3
......................uggaucuuguuugacagcaAuc	1	1	JCK-2
......................uggaucuuguuugacagcacuU	27	1	LT-2
......................uggaucuuguuugacagcGcuc	3	1	JCK-2
......................Cggaucuuguuugacagcacuc	1	1	LT-1
......................uggaucuuguuuUacagcacuc	1	1	JCK-3
......................uggaucuuguuugacagcGcuc	1	1	JCK-1
......................uggaucuuguuugacaCcacuc	1	1	JCK-3
......................uggaucuuguuuAacagcacuc	1	1	JCK-1
......................uggaucuuguuugacagcacucA	100	1	JCK-3
......................uggaucuuguuugacagcacuUu	62	1	LT-1
......................uggaucuuguuugacagcacuGu	2	1	LT-2
......................uggaucuuguuugacagcacucA	23	1	LT-1
......................uggCucuuguuugacagcacucu	1	1	LT-2
......................uggaucuuguuugacagcacuUu	159	1	JCK-2
......................uggaucuuguuugacagcacuUu	21	1	JT-2
......................uggaucuuguuugacagcacucG	5	1	JCK-2
......................uggaucuuguuugacagcacucu	26	0	LT-1
......................uggaucuuguuugacagcacucA	14	1	JT-3
......................uggaucuuguuugacagcacucu	80	0	JCK-3
......................uggaucuuguuugacagcacuUu	75	1	LCK-3
......................uggaucuuguuugacagcacucA	10	1	JT-2
......................uggaucuuguuugacagcacuAu	1	1	JCK-3
......................uggaucuuguuugacagcacucu	111	0	JCK-2
......................uggaucuuguuugacagcacuUu	71	1	LT-2
......................uggaucuuguuugacagcacucA	79	1	JCK-1
......................uggaucuuguuugacagcacucu	90	0	JCK-1
......................uggaucuuguuNgacagcacucu	3	1	JCK-1
......................uggaucuuguuugacagcacucu	16	0	LCK-3
......................uggaucuuguuugacagcacucu	14	0	LT-2
......................uggaucuuguuugacagcacucA	24	1	LCK-3
......................uggaucuuguuugacagcacucA	14	1	LT-2
......................uggaucuuguuugacagcacucA	6	1	LT-3
......................uggaucuuguuugacagcacucu	10	0	LT-3
......................uggaucuuguuugacagcacuAu	2	1	JCK-2
......................uggaucuuguuugacagcacuAu	3	1	JCK-1
......................uggaucuuguuugacagcacucG	3	1	JCK-1
......................uggaucuuguuugacagcacucG	4	1	JCK-3
......................uggaucuuguuugacagcacucA	140	1	JCK-2
......................uggaucuuguuugacagcacuGu	5	1	LT-1
......................uggaucuuguuugacagcacuUu	112	1	JCK-3
......................uggaucuuguuugacagcacuUu	109	1	JCK-1
......................uggaucuuguuugacagcacuUu	39	1	JT-1
......................uggaucuuguuugacagcacuGu	6	1	JCK-2
......................uggaucuuguuugacagcacuUu	23	1	LCK-1
......................uggaucuuguuugacagcacuUu	21	1	JT-3
......................uggaucuuguuugacagcacuGu	1	1	LT-3
......................uggaucuuguuugacagcacuUu	19	1	LCK-2

```
                     Mature                                                                         Star
uccaucuuguucguuuacccucuggaucuuguuugacagcacucucuuauauuuugccauucaucuuuauaugccaaauggcgaugaugaugauggugga gugcuaucgacaugauccagaaggugcagucgaauaagauacagagggaccacccacaguaccuacucucguuacaaucguauucuucuccucacuuauuauuauuauuauuauuauuccgaucauuuucuuuuaaucuauugaag
......................uggaucuuguuugacagcacuGu.........................................................................................................................................................................................................   1   1   JCK-1
......................uggaucuuguuugacagcacuUu.........................................................................................................................................................................................................   53  1   LT-3
......................uggaucuuguuugacagcacuUuc........................................................................................................................................................................................................   3   1   JCK-2
......................uggaucuuguuugacagcacucuc........................................................................................................................................................................................................   19  0   JCK-3
......................uggaucuuguuugacagcacucAc........................................................................................................................................................................................................   7   1   LT-1
......................uggaucuuguuugacagcacucAc........................................................................................................................................................................................................   2   1   LT-3
......................uggaucuuguuugacagcacucuA........................................................................................................................................................................................................   2   1   JCK-2
......................uggaucuuguuugacagcacucuA........................................................................................................................................................................................................   5   1   LT-2
......................uggaucuuguuugacagcacucuU........................................................................................................................................................................................................   9   1   LT-1
......................uggaucuuguuugacagcacucuc........................................................................................................................................................................................................   3   0   LCK-3
......................uggaucuuguuugacagcacucuA........................................................................................................................................................................................................   2   1   JCK-3
......................uggaucuuguuugacagcacucAc........................................................................................................................................................................................................   13  1   LCK-3
......................uggaucuuguuugacagcacucuc........................................................................................................................................................................................................   1   0   LT-3
......................uggaucuuguuugacagcacucuc........................................................................................................................................................................................................   12  0   JCK-1
......................uggaucuuguuugacagcacucuA........................................................................................................................................................................................................   2   1   JCK-1
......................uggaucuuguuugacagcacucuU........................................................................................................................................................................................................   7   1   LT-2
......................uggaucuuguuugacagcacucuc........................................................................................................................................................................................................   19  0   JCK-2
......................uggaucuuguuugacagcacucuA........................................................................................................................................................................................................   1   1   LT-1
......................uggaucuuguuugacagcacucuU........................................................................................................................................................................................................   1   1   JCK-1
......................uggaucuuguuugacagcacuGuc........................................................................................................................................................................................................   1   1   LT-3
......................uggaucuuguuugacagcacucuU........................................................................................................................................................................................................   6   1   JCK-2
......................uggaucuuguuugacagcacucuA........................................................................................................................................................................................................   1   1   LT-3
......................uggaucuuguuugacagcacucuc........................................................................................................................................................................................................   1   0   LT-1
......................uggaucuuguuNgacagcacucuc........................................................................................................................................................................................................   1   1   JCK-1
......................uggaucuuguuugacagcacucucu.......................................................................................................................................................................................................   2   0   JCK-3
......................uggaucuuguuugacagcacucAcu.......................................................................................................................................................................................................   1   1   JCK-2
......................uggaucuuguuugacagcacucuUu.......................................................................................................................................................................................................   1   1   LT-1
......................uggaucuuguuugacagcacucuUu.......................................................................................................................................................................................................   6   1   LT-2
......................uggaucuuguuugacagcacucucu.......................................................................................................................................................................................................   1   0   LT-3
......................uggaucuuguuugacagcacucuUu.......................................................................................................................................................................................................   1   1   JCK-1
......................uggaucuuguuugacagcacucuAu.......................................................................................................................................................................................................   1   1   LT-1
......................uggaucuuguuugacagcacucuUu.......................................................................................................................................................................................................   2   1   JCK-2
......................uggaucuuguuugacagcacucucu.......................................................................................................................................................................................................   6   0   JCK-2
......................uggaucuuguuugacagcacucucu.......................................................................................................................................................................................................   1   0   JCK-1
......................uggaucuuguuugacagcacucuAuu......................................................................................................................................................................................................   1   1   JCK-1
.......................ggaucuuguuugacagca.............................................................................................................................................................................................................   2   0   JCK-3
.......................ggaucuuguuugacagca.............................................................................................................................................................................................................   1   0   JCK-1
.......................ggaucuuguuugacagca.............................................................................................................................................................................................................   1   0   LT-1
.......................ggaucuuguuugacagcac............................................................................................................................................................................................................   1   0   LT-2
.......................ggaucuuguuugacagcac............................................................................................................................................................................................................   4   0   LCK-3
.......................ggaucuuguuugacagcac............................................................................................................................................................................................................   2   0   LT-1
.......................ggaucuuguuugacagcacu...........................................................................................................................................................................................................   3   0   JCK-2
.......................ggaucuuguuugacagcacu...........................................................................................................................................................................................................   2   0   LT-3
.......................ggaucuuguuugacagcacu...........................................................................................................................................................................................................   5   0   LT-2
.......................ggaucuuguuugacagcacu...........................................................................................................................................................................................................   2   0   LCK-3
.......................ggaucuuguuugacagcacu...........................................................................................................................................................................................................   5   0   JCK-1
.......................ggaucuuguuugacagcacu...........................................................................................................................................................................................................   5   0   LT-1
.......................ggaucuuguuugacagcacu...........................................................................................................................................................................................................   4   0   JCK-3
.......................ggaucuuguuugacagcacuc..........................................................................................................................................................................................................   14  0   JCK-3
.......................ggaucuuguuugacagcacuc..........................................................................................................................................................................................................   6   0   JCK-1
.......................ggaucuuguuugacagcacuc..........................................................................................................................................................................................................   6   0   JCK-2
.......................ggaucuuguuugacagcacucu.........................................................................................................................................................................................................   5   0   JCK-1
.......................ggaucuuguuugacagcacucA.........................................................................................................................................................................................................   2   1   JCK-2
.......................ggaucuuguuugacagcacucu.........................................................................................................................................................................................................   6   0   JCK-2
.......................ggaucuuguuugacagcacucu.........................................................................................................................................................................................................   4   0   JCK-3
.......................ggaucuuguuugacagcacucucu.......................................................................................................................................................................................................   2   0   JCK-2
........................gaucuuguuugacagcacu...........................................................................................................................................................................................................   3   0   LT-3
........................gaucuuguuugacagcacu...........................................................................................................................................................................................................   14  0   LT-2
........................gaucuuguuugacagcacu...........................................................................................................................................................................................................   1   0   LT-1
........................gaucuuguuugacagcacu...........................................................................................................................................................................................................   3   0   JCK-1
........................gaucuuguuugacagcacu...........................................................................................................................................................................................................   1   0   JCK-2
........................gaucuuguuugacagcacuc..........................................................................................................................................................................................................   2   0   LT-1
........................gaucuuguuugacagcacuc..........................................................................................................................................................................................................   3   0   LT-3
........................gaucuuguuugacagcacucA.........................................................................................................................................................................................................   1   1   LT-1
........................gaucuuguuugacagcacucu.........................................................................................................................................................................................................   1   0   LT-1
........................gaucuuguuugacagcacucuc........................................................................................................................................................................................................   4   0   JCK-2
........................gaucuuguuugacagcacucuc........................................................................................................................................................................................................   1   0   JCK-1
........................gaucuuguuugacagcacucuc........................................................................................................................................................................................................   1   0   JCK-3
.........................aucuuguuugacagcacu...........................................................................................................................................................................................................   3   0   LT-2
.........................aucuuguuugacagcacu...........................................................................................................................................................................................................   2   0   LT-1
```

```
                      Mature                                                                               Star
uccaucuuguucguuuacccucuggaucuuguuugacagcacucucuuauauuuugccauucaucuuuauaugccaaauggcgaugaugaugaugguggga gugcuaucgacaugauccagaaggugcagucgaauaagauacagagggaccacccacaguaccuacucucguuacaaucguauucuucuccucacuuauuauuauuauuauuauuauuccgaucauuuucuuuuaaucuauugaag
.........................aucuuguuugacagcacuc............................................................................................................................................................................................................  8  0  LCK-3
.........................aucuuguuugacagcacuc............................................................................................................................................................................................................  1  0  LT-2
.........................aucuuguuugacagcacuc............................................................................................................................................................................................................  2  0  LT-1
.........................aucuuguuugacagcacuc............................................................................................................................................................................................................  1  0  JCK-2
.........................aucuuguuugacagcacuc............................................................................................................................................................................................................  5  0  LT-3
.........................aucuuguuugacagcacuU............................................................................................................................................................................................................  1  1  LT-2
.........................aucuuguuugacagcacucA...........................................................................................................................................................................................................  7  1  LCK-3
.........................aucuuguuugacagcacucu...........................................................................................................................................................................................................  4  0  JCK-3
.........................aucuuguuugacagcacucA...........................................................................................................................................................................................................  2  1  LT-1
.........................aucuuguuugacagcacucA...........................................................................................................................................................................................................  1  1  LT-2
.........................aucuuguuugacagcacuUu...........................................................................................................................................................................................................  1  1  LT-3
.........................aucuuguuugCcagcacucu...........................................................................................................................................................................................................  1  1  JCK-2
.........................aucuuguuugacagcacucu...........................................................................................................................................................................................................  15  0  JCK-2
.........................aucuuguuugacagcacucu...........................................................................................................................................................................................................  4  0  LT-1
.........................aucuuguuugacagcacucu...........................................................................................................................................................................................................  2  0  LT-2
.........................aucuuguuugacagcacucu...........................................................................................................................................................................................................  3  0  JCK-1
.........................aucuuguuugacagcacucu...........................................................................................................................................................................................................  24  0  LCK-3
.........................aucuuguuugacagcacuUu...........................................................................................................................................................................................................  1  1  LT-1
.........................aucuuguuugacagcacucu...........................................................................................................................................................................................................  4  0  LT-3
.........................aucuuguuugacagcacucuA..........................................................................................................................................................................................................  1  1  JCK-3
.........................aucuuguuugacagcacucuU..........................................................................................................................................................................................................  4  1  LT-2
.........................Cucuuguuugacagcacucuc..........................................................................................................................................................................................................  1  1  JCK-2
.........................aucuuguuugacagcacucuA..........................................................................................................................................................................................................  3  1  JCK-1
.........................aucuuguuugacagcacucuc..........................................................................................................................................................................................................  8  0  JCK-1
.........................aucuuguuugacagcacucuU..........................................................................................................................................................................................................  6  1  LCK-3
.........................aucuuguuugacagcacucuc..........................................................................................................................................................................................................  12  0  JCK-2
.........................aucuuguuugacagcacucuc..........................................................................................................................................................................................................  11  0  JCK-3
.........................aucuuguuugacagcacucCcu.........................................................................................................................................................................................................  1  1  JCK-2
.........................aucuuguuugacGgcacucucu.........................................................................................................................................................................................................  1  1  JCK-3
.........................aucuuguuugacagcacucCcu.........................................................................................................................................................................................................  1  1  LT-3
.........................aucuuguuugacagcacucucu.........................................................................................................................................................................................................  1  0  LT-1
.........................aucuuguuugacagcacucucu.........................................................................................................................................................................................................  70  0  JCK-1
.........................aucuuguuugacagcacucucu.........................................................................................................................................................................................................  92  0  JCK-3
.........................Cucuuguuugacagcacucucu.........................................................................................................................................................................................................  1  1  JCK-1
.........................aucuuguuugCcagcacucucu.........................................................................................................................................................................................................  1  1  JCK-1
.........................aucuuguuugacagcacucCcu.........................................................................................................................................................................................................  1  1  JCK-3
.........................aucuuguuugacagcacucCcu.........................................................................................................................................................................................................  1  1  JCK-1
.........................aucuuguuugacagcacucucu.........................................................................................................................................................................................................  107  0  JCK-2
.........................aucuuguuugacagcacucucG.........................................................................................................................................................................................................  1  1  JCK-1
.........................aucuuguuugacagcacucucuA........................................................................................................................................................................................................  1  1  JCK-1
.........................aucuuguuugacagcacucucuA........................................................................................................................................................................................................  1  1  JCK-2
.........................aucuuguuCgacagcacucucuu........................................................................................................................................................................................................  1  1  JCK-1
.........................aucuuguuugacagcacucucuuaAau....................................................................................................................................................................................................  1  1  JCK-2
.........................aucuuguuugacagcacucucuuaAau....................................................................................................................................................................................................  1  1  JCK-1
..........................ucuuguuugacagcacuc............................................................................................................................................................................................................  1  0  LT-2
..........................ucuuguuugacagcacuc............................................................................................................................................................................................................  2  0  LT-3
..........................ucuuguuugacagcacucu...........................................................................................................................................................................................................  2  0  LT-1
..........................ucuuguuugacagcacucA...........................................................................................................................................................................................................  1  1  LT-3
..........................ucuuguuugacagcacucu...........................................................................................................................................................................................................  2  0  JCK-2
..........................ucuuguuugacagcacucu...........................................................................................................................................................................................................  2  0  JT-2
..........................ucuuguuugacagcacucuc..........................................................................................................................................................................................................  2  0  JCK-2
..........................ucuuguuugacagcacucuc..........................................................................................................................................................................................................  1  0  JCK-3
..........................ucuuguuugacagcacucucu.........................................................................................................................................................................................................  13  0  JCK-2
..........................ucuuguuugacagcacucucu.........................................................................................................................................................................................................  1  0  LT-1
..........................ucuuguuugacagcacucucu.........................................................................................................................................................................................................  2  0  LT-3
..........................ucuuguuugacagcacucucu.........................................................................................................................................................................................................  6  0  JCK-3
..........................ucuuguuugacagcacucucu.........................................................................................................................................................................................................  3  0  JCK-1
..........................ucuuguuugacagcacucucuCa.......................................................................................................................................................................................................  1  1  LT-1
...........................................................................aaauggUgaugaugaugaug.........................................................................................................................................................  2  1  JCK-3
.............................................................................auggcgaugaAgaugaug.........................................................................................................................................................  1  1  LT-1
..............................................................................................gAugggagugcuaucgacaugauc..................................................................................................................................  2  1  LT-1
...............................................................................................Augggagugcuaucgacaugau...................................................................................................................................  1  1  LT-3
.................................................................................................gggagugcuGucgacaugauccagaaggug.........................................................................................................................  1  1  JCK-3
....................................................................................................agugcuaucgacaugauccaga..............................................................................................................................  1  0  LT-3
.....................................................................................................gugcuaucgaAaugaucca................................................................................................................................  1  1  JCK-2
.....................................................................................................gugcuaucgacaugaucca................................................................................................................................  1  0  LT-1
.....................................................................................................gugcuaucgacaugauccag...............................................................................................................................  2  0  LT-1
.....................................................................................................gugcuaucgacaugauccag...............................................................................................................................  1  0  JCK-1
.....................................................................................................gugcuaucgacaugauccag...............................................................................................................................  1  0  LT-2
.....................................................................................................gugcuaucgacaugauccaga..............................................................................................................................  6  0  JCK-1
```

```
                    Mature                                                                      Star
uccaucuuguucguuuacccucuggaucuuguuugacagcacucucuuauauuuugccauucaucuuuauaugccaaauggcgaugaugaugauggugggagugcuaucgacaugauccagaaggugcagucgaauaagauacagagggaccacccacaguaccuacucucguuacaaucguauucuucuccucacuuauuauuauuauuauuauuauuauuccgaucauuuucuuuuaaucuauugaag
.....................................................................................................gugcuaucgacaugauccaga................................................................................................................................   36   0   LCK-3
.....................................................................................................gugcuaucgacaugauccaga................................................................................................................................   12   0   LT-2
.....................................................................................................gugcuaucgacaugauccagC................................................................................................................................   1    1   LT-2
.....................................................................................................gugcuaucgacaugauccaga................................................................................................................................   3    0   JCK-3
.....................................................................................................gugcuaucgacaugauccaga................................................................................................................................   6    0   LT-1
.....................................................................................................gugcuaucgacaugauccaga................................................................................................................................   14   0   LT-3
.....................................................................................................Augcuaucgacaugauccaga................................................................................................................................   1    1   JCK-2
.....................................................................................................gugcuaucgacaugauccaga................................................................................................................................   4    0   JCK-2
......................................................................................................ugcuaucgacCugauccag.................................................................................................................................   1    1   LT-1
......................................................................................................ugcuaucgacaugauccag.................................................................................................................................   2    0   LT-1
......................................................................................................ugcuaucgacaugauccaga................................................................................................................................   6    0   JCK-3
......................................................................................................ugcuaucgacaugauccaga................................................................................................................................   5    0   JCK-1
......................................................................................................ugcuaucgacaugauccaga................................................................................................................................   2    0   LT-3
......................................................................................................ugcuaucgacaugauccaga................................................................................................................................   16   0   JCK-2
......................................................................................................uNcuaucgacaugauccaga................................................................................................................................   1    1   JCK-1
............................................................................................................ucgacaAgauccagaaggugc.........................................................................................................................   2    1   JCK-1
............................................................................................................ucgacaAgauccagaaggugc.........................................................................................................................   3    1   JCK-3
............................................................................................................ucgacaAgauccagaaggugc.........................................................................................................................   1    1   JCK-2
```
